# Emergence of kinship structures and descent systems: multi-level evolutionary simulation and empirical data analysis

**DOI:** 10.1098/rspb.2021.2641

**Published:** 2022-02-23

**Authors:** Kenji Itao, Kunihiko Kaneko

**Affiliations:** ^1^ Department of Basic Science, Graduate School of Arts and Sciences, University of Tokyo, Komaba 3-8-1, Meguro-ku, Tokyo 153-8902, Japan; ^2^ Research Center for Complex Systems Biology, University of Tokyo, Komaba 3-8-1, Meguro-ku, Tokyo 153-8902, Japan

**Keywords:** multi-level evolution, agent-based modelling, cultural evolution, kinship structure, descent system, universal anthropology

## Abstract

In many indigenous societies, people are categorized into several cultural groups, or clans, within which they believe they share ancestors. Clan attributions provide certain rules for marriage and descent. Such rules between clans constitute kinship structures. Anthropologists have revealed several kinship structures. Here, we propose an agent-based model of indigenous societies to reveal the evolution of kinship structures. In the model, several societies compete. Societies themselves comprise multiple families with parameters for cultural traits and mate preferences. These values determine with whom each family cooperates and competes, and they are transmitted to a new generation with mutation. The growth rate of each family is determined by the number of cooperators and competitors. Through this multi-level evolution, family traits and preferences diverge to form clusters that can be regarded as clans. Subsequently, kinship structures emerge, including dual organization and generalized or restricted exchange, as well as patrilineal, matrilineal and double descent systems. These structures emerge depending on the necessity of cooperation and the strength of mating competition. Their dependence is also estimated analytically. Finally, statistical analysis using the Standard Cross-Cultural Sample, a global ethnographic database, empirically verified the theoretical results. Such collaboration between theoretical and empirical approaches will unveil universal features in anthropology.

## 1. Introduction

Marriage and descent shape the basic units of society, that is, families. Kinship relationships, including marriage, descent and cultural relatedness, stipulate the alliance of families and organize social structures. It is considered as one of the oldest, most frequent human social organizations [1,2]. In various indigenous societies, people constitute a cultural association, or clan, in which they are regarded as cultural (but not necessarily biological) kin [1,3,4]. Kinship relationships are determined by clan attributions (i.e. the clan to which individuals belong). Specifically, marriage within a clan is often prohibited by the symbolic incest taboo [1,5–9]. The rule can further specify the clan from which one must select a mate and that to which children must belong [1]. Additionally, kinship relationships regulate other social relationships, such as cooperation or rivalry [3]. The elucidation of kinship systems has been a core theme in cultural and evolutionary anthropology [3,10]. Anthropologists have characterized kinship systems by focusing on the affinal network of clans, namely, kinship structure [1]; or by the categorization of relatives by ego, namely, kinship terminology [7,11]. Here, we consider kinship structures.

Kinship structures are diverse yet patterned. They can be classified into several types, according to the length of cycles composed by the marriage and descent relationships of clans [1,12]. For example, if a rule exists for women in clan X to marry men in clan Y, the marriage relationship is represented by X ⇒ Y. If everyone can potentially have mates, the relationships of clans should be X ⇒ Y ⇒ · · · ⇒ X. Here, the marriage relationships of clans constitute a cycle, the length of which is termed marriage cycle *C*_*m*_ (e.g. *C*_*m*_ = 3 if X ⇒ Y ⇒ Z ⇒ X). Similarly, if the children belong to clan B, and their father to clan A, it represents the descent relationship A → B. This relationship also constitutes the cycle, and its length is termed the descent cycle *C*_*d*_. Notably, a clan is not always a residence group. Family members of different generations can have different clan attributions [13]. For example, when children inherit their father’s surname but live in their mother’s location, the children’s attribution, determined by both surname and location, differs from those of both their father and mother. (This can also be regarded as children belonging to several associations following each parent simultaneously [2].)

Kinship structures are characterized by marriage cycle *C*_*m*_ and descent cycle *C*_*d*_. The classes include clan endogamy—without the symbolic incest taboo (*C*_*m*_ = *C*_*d*_ = 1, note that clans may not be divided); dual organization—a direct exchange of brides between two clans (*C*_*m*_ = 2 and *C*_*d*_ = 1); generalized exchange—an indirect exchange of brides among more than two clans (*C*_*m*_ ≥ 3 and *C*_*d*_ = 1); and restricted exchange—a direct exchange of brides with the flow of children to different clans (*C*_*m*_ = *C*_*d*_ = 2). Structures with *C*_*m*_ ≥ 3 and *C*_*d*_ ≥ 2 are rarely observed.

In this paper, we discuss the evolution of three types of descent systems. When children belong to the same clan as their father (or mother), the descent system is classified as patrilineal (or matrilineal) descent. In these cases, *C*_*d*_ = 1, and the paternally (or maternally) inherited trait is significant for characterizing clans. Conversely, when *C*_*d*_ > 1, children inherit cultural traits from both parents independently and have clan attributions different from either parent. When both paternally and maternally inherited traits are significant for characterizing clans, the system is termed double descent. In the above cases, traits are assumed to be independently inherited through paternal and maternal lines. (In some societies, however, people can choose either their father’s or mother’s traits to inherit in each generation (ambilineal descent) or they concern genealogical distance only (bilateral descent), which exceeds the scope of our model [2,14].)

Ethnographic reports provide examples of various descent systems [1,15,16]. Global data indicate that patrilineal descent is more common than matrilineal or double descent [17]. Evolutionary anthropologists attribute this imbalance to the higher investment efficiency of reproductive resources for sons than for daughters [10,18,19]. However, this perspective ignores that people are categorized as clans by symbolic traits inherited through some descent lines. Indeed, the identity of the categorical descent group is strongly emphasized (more than genetic relatedness) in some cooperative actions [20,21].

Moreover, cultural traits of family and kinship are slow to change because they are inherited in families and regulated by social norms [22]. Empirical studies confirm such slow changes [23–25]. To consider the inheritance of family traits from parents or their relatives with slight changes, it is appropriate to model their long-term evolution through the accumulation of small variations, as represented by mutations. Notably, families constitute society, whereas society provides the environment for families. Consequently, we adopted a framework involving the multi-level evolution of families and societies. Multi-level evolution is a framework generally applied for discussing the evolution of group-level structures in hierarchical systems [26–29]. In this study, we aimed to reveal the emergence of various kinship structures and descent systems from family interactions depending on environmental conditions.

We thus modelled the family behaviour that is common in traditional indigenous societies. In the model, evolution is considered at two levels: that of the family, which is an individual agent of the model; and that of society, which is a group of families. We assigned each family a trait *t* and a mate preference *p*. Social relationships of families—including cooperation, competition and marriage—are determined by their traits and preferences. Families grow through interactions with other families, which subsequently lead to the growth of societies. As a result of this multi-level evolutionary simulation, *t*, *p* values of families diverge and form clusters within each society. These clusters are exogamous groups of families, which can be regarded as clans. By tracing the marriage and descent relationships of the emergent clans, we demonstrate the evolution of kinship structures and descent systems. Previously, we had constructed an intricate model to illustrate the evolution of kinship structures [30]. Here, we introduce a simplified model suitable for studying the evolution of both kinship structures and descent systems, together with analytical estimates and empirical tests on a cross-cultural database.

For data analysis, we used the global ethnographic database of premodern societies, the Standard Cross-Cultural Sample (SCCS) [17,31]. The SCCS contains 186 societies, considered culturally and linguistically independent of each other (even if some correlation exists due to the shared ancestry in the strict sense [25]). The data allowed us to quantitatively analyse cultural adaptations to environments [32,33]. Previous studies have investigated conditions that generally favour cousin marriages [34] and polygamy [35]. However, it is difficult to further explain the diversity in cousin marriage and kinship structures solely from correlation analyses [36]. Thus, we demonstrate that the collaboration between theoretical simulation and statistical analysis can enable us to suggest the origins of, and conditions for, each kinship structure.

The remainder of this paper is organized as follows. In the next section, we introduce a simplified model. Then, using evolutionary simulations, we demonstrate the emergence of kinship structures and descent systems, and uncover the conditions for their emergence. We also estimate these conditions analytically. Next, by analysing the SCCS, the theoretical results are verified. Finally, we discuss how the present method, which combines theoretical models and empirical data analysis, is relevant to exploring anthropological phenomena.

## 2. Model

**Figure 1.**
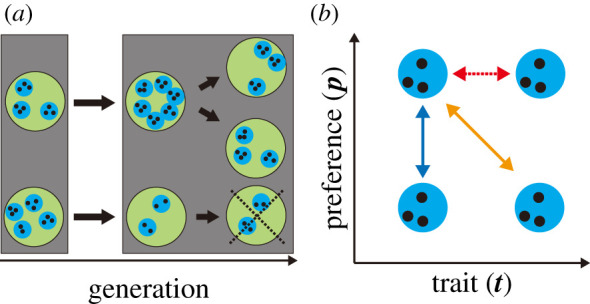
Schematic of the model. (*a*) Life cycle of the model. Societies (green) consist of families (blue), whose population (black) grows. The grey frame represents a single generation. Families grow through interactions with other families in the same society. When the population of a society exceeds a given threshold, the society splits. Subsequently, another society is removed from the system at random to keep the number of societies fixed. (*b*) Families cooperate with kin and mates (blue and orange solid lines), and conflict with rivals (red dashed line) depending on their traits ***t*** and mate preferences ***p***. Families *i* and *j* are kin (blue) when |***t***^*i*^ − ***t***^*j*^| is sufficiently small, mates (orange) when |***t***^*i*^ − ***p***^*j*^| or |***p***^*i*^ − ***t***^*j*^| is small, and rivals (red) when |***p***^*i*^ − ***p***^*j*^| is small. (Note that some families can be rivals and kin/mates simultaneously.) Only the relationships with the upper-left family are shown. In the figure, we plotted the relationships in a two-dimensional space for simplicity. In the model, however, ***t*** and ***p*** are both two-dimensional. Thus, the relationships are considered in four-dimensional space. (Online version in colour.)

The model is described below in general terms (see §6 for further details). Figure 1 shows a schematic of our model. Families grow by interacting with other families in the same society (figure 1*a*). Here, we ignore the explicit interaction between societies including migration, for simplicity. However, the following results are robust for slight migrations, as shown in electronic supplementary material, figure S1. At the time of marriage, family members independently build new families of their own. The society splits in half when the number of families therein doubles its initial value *N*_*f*_. At this time, another society is removed at random; thus, the number of societies in the entire system remains fixed at *N*_*s*_. However, the number of families fluctuates between 0 and 2*N*_*f*_. This process introduces society-level selection, such that societies that grow at a faster rate replace others. This can be interpreted as an invasion, imitation or the coarse-grained description of a growing system. This framework, known as the multi-level selection, has been widely adopted in biological and social evolution studies to explain group-level structures [27–30,37–39]. Previously, we considered a model with three layers, including the intermediate layer of ‘lineage’ between family and society. Here, we simplify the model by eliminating lineages, to explore the generality of the results and to enable suitable analytical calculations [30].

Moreover, each family has a pair of cultural traits and mate preferences that are culturally transmitted to the next generation. The traits can represent any social features by which people can measure their cultural similarity, for instance, surnames, occupations or totems [40]. In the following section, we demonstrate that initially uniform traits gradually diverge to be discrete for distinguishing family groups. Marriage occurs when men’s traits are close to women’s preferences. In our model, this point is the sole asymmetry between men and women. Note that anthropological studies state that brides’ families determine whether grooms are suitable for marriage in many societies [1,40].

There are two pathways for cultural transmission: paternal and maternal. Hence, we require the two-dimensional trait ***t*** = (*t*_1_, *t*_2_) and preference ***p*** = (*p*_1_, *p*_2_). Thus, when a man in family *i* and a woman in family *j* are married, their children will have the trait $t=(t_{1}^{i},t_{2}^{j})$ and preference $p=(p_{1}^{i},p_{2}^{j})$. At the time of cultural transmission, we add noise ***η*** = (*η*_1_, *η*_2_) to ***t*** and ***p***, independently sampled from a normal distribution with mean 0 and variance *μ*^2^. Similar to genetic mutations in evolutionary biology, cultural traits are slightly modified when they are transmitted [22]. Such cultural traits are used to categorize social groups, even without genetic relatedness [41]. Previously, we assumed that *t*_1_ and *p*_1_ are inherited from the father, and *t*_2_ and *p*_2_ are inherited either from the father or the mother, depending on the families’ strategies [30]. However, this assumption limits the evolution of descent systems, as the matrilineal descent system is set to be harder to evolve. Here, we revised this to enable discussion on the evolution of various descent systems. (Notably, paternal and maternal traits are still assumed to be inherited independently. Hence, those descent systems in which both parents’ traits are multiply referenced exceed the scope of our model.)

First, we introduced cooperative relationships with cultural kin and mates (blue and orange solid lines in figure 1*b*). Families cooperate with those who have traits similar to their own, and those who prefer (or are preferred by) them. In the model, the degree of cooperation between family *i* and *j* is given by exp(−min(|***t***^*i*^ − ***t***^*j*^|, |***t***^*i*^ − ***p***^*j*^|, |***p***^*i*^ − ***t***^*j*^|)^2^/*τ*^2^), where $|t^{i}-t^{j}|=\sqrt{{\left(t_{1}^{i}-t_{1}^{j}\right)}^{2}+{\left(t_{2}^{i}-t_{2}^{j}\right)}^{2}}$ represents Euclidean distance and *τ* represents the tolerance for similar traits and preferences. By averaging this degree for families in the same society, we calculated the density of cooperative families *friend*_*i*_ for each family *i*. A smaller *friend* value implies that the family gains less cooperation, resulting in a decline in the growth rate, where *d*_*c*_ represents the death rate increment due to non-cooperation.

Next, we introduced competitive relationships with mating rivals (red dashed line in figure 1*b*). Families compete with those who have similar preferences. The degree between families *i* and *j* is given by exp(−|***p***^*i*^ − ***p***^*j*^|^2^/*τ*^2^). We calculated the density of competitive families *rival*_*i*_ for each family *i*. A larger *rival* value implies that the family has many rivals, resulting in a decline in the growth rate, where *d*_*m*_ represents an increase in the death rate owing to competition. Here, the strength of competition depends only on the number of families with close preferences. It is independent of the number of preferred families because competition occurs even when there are sufficient grooms and brides [42].

Then, we calculated the population growth as determined by the interactions of families. The number of men and women in family *i* who survive until marriageable age is given by Poisson distribution with mean *b*exp(− *d*_*c*_(1− *friend*_*i*_) − *d*_*m*_ *rival*_*i*_), where *b* determines the intrinsic growth rate. We adopted this form because it is more suitable for analytical calculations. The presented results are qualitatively independent of the specific forms. For example, *b*− *d*_*c*_(1− *friend*) − *d*_*m*_ *rival* or *b*/((1 + *d*_*c*_(1− *friend*))(1 + *d*_*m*_ *rival*)) (the latter was adopted in the previous model [30]) essentially produces identical results if cooperation enhances, and conflict suppresses, the population.

Finally, people get married according to their traits and preferences. The probability of marriage of men in family *i* and women in family *j* is proportional to exp(− |***t***^*i*^− ***p***^*j*^|^2^/*τ*^2^). After marriage, couples create their own families, bear children who inherit traits and preferences, and then die.

The initial values of ***t***, ***p*** are (0, 0) in this model. Thus, at first, no rules concerning marriage or descent existed. Initially, any couple could marry, even within a nuclear family. This assumption is set to demonstrate that society-level structures, which determine the marriage rules of families, can evolve, even when starting from an initially homogeneous state. However, the results after sufficient generations are independent of the initial conditions. The notations and parameter values adopted in the simulations are summarized in table 1.

**Table 1.** Parameters used in the model. In the results described below, the values of *b*, *μ*, *τ*, *N*_*f*_ and *N*_*s*_ are fixed to those shown in the table, unless the value is described explicitly.

sign	explanation	value
*b*	intrinsic growth rate	5.0
*μ*	mutation rate for ***t***, ***p***	0.1
*τ*	tolerance for similarity	1.0
*N*_*f*_	initial number of families in society	50
*N*_*s*_	number of societies in a system	50
*d*_*c*_	decline in mortality with cooperation	variable
*d*_*m*_	increase in mortality with competition	variable
***t***	cultural traits of family	evolve
***p***	preferences for groom traits	evolve

## 3. Evolution of kinship structures

**Figure 2.**
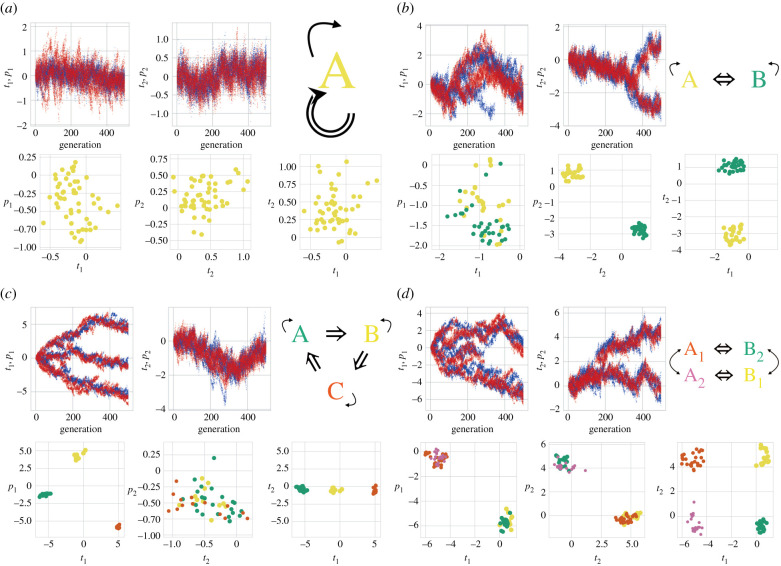
Examples of the evolution of kinship structures. (***t***, ***p***) values of families in society after 500 simulation steps. The figures show the temporal evolution of (***t***, ***p***) values (upper-left), a schematic of the emergent structure (upper-right) and the final state (bottom). The temporal evolution of the trait and preference values of families in a society are represented in blue and red, respectively. The final states are shown as a *t*_1_− *p*_1_ map, a *t*_2_− *p*_2_ map and a *t*_1_− *t*_2_ map, from left to right. (The scales of axes differ, depending on the variance of values.) The structures are categorized by calculating the marriage (*C*_*m*_) and descent cycles (*C*_*d*_) as the lengths of the cycles of the flow of women and children, respectively. (*a*) Clan endogamy without the division of clans. Marriage occurs within clan A (yellow). *d*_*c*_ = 5.0, *d*_*m*_ = 0.1. (*b*) Dual organization with a matrilineal descent system. Clans A (yellow) and B (green) diverge concerning the maternally inherited trait *t*_2_ and prefer each other. *d*_*c*_ = 0.3, *d*_*m*_ = 0.2. (*c*) Generalized exchange with a patrilineal descent system. Clans A (green), B (yellow) and C (orange) diverge concerning the paternally inherited trait *t*_1_ and prefer others cyclically. *d*_*c*_ = 0.5, *d*_*m*_ = 1.0. (*d*) Restricted exchange with a double descent system. Clans A_1_ (orange), A_2_ (pink), B_1_ (yellow) and B_2_ (green) exhibit pairwise marriage and descent relationships. Here, clans diverge regarding both maternally and paternally inherited traits. *d*_*c*_ = 0.2, *d*_*m*_ = 1.0. (Online version in colour.)

The model was simulated iteratively for various parameter values listed in table 1. In a simulation of 500 steps, the (***t***, ***p***) values of families within a society diverged, and finally, formed some clusters in (***t***, ***p***) space, as shown in figure 2. With the pressure to increase cooperators by increasing kin and mates, isolated families without sufficient *friend* values are removed, and as a result, families’ traits are clustered and families prefer others’ traits. With the pressure to decrease mating rivals, families’ preferences diverge. Accordingly, under sufficient strengths of both pressures, that is, sufficient *d*_*c*_ and *d*_*m*_ values, families form several discrete clusters united by marital relationships in (***t***, ***p***) space. Siblings belonged to the same cluster at birth. Families within the same cluster, including those who were genetically unrelated, had similar traits and recognized each other as cultural kin. They avoided marriage within their cluster and preferred mates from other clusters, that is, $t^{i}≄p^{i}$ to increase cooperators by acquiring mates other than their cultural kin. Consequently, the emergent clusters were culturally united groups with the symbolic incest taboo, preferring exogamy. They can, therefore, be interpreted as clans. Clans were attributed based on parental traits. Here, the different clans are characterized by discretized trait values. Discretization for *t*_1_, *t*_2_ or both values leads to the evolution of various descent systems. In this model, clans’ descent relationships, as well as their marriage relationships, emerged. The relationships between clans were determined by tracing the marriage and descent relationships of the cluster centres. Here, we used the *X*-means method for clustering to optimize the number of clusters by adopting the Bayesian information criterion [43]. The emergent structures were classified according to the cycles of marriage and descent relationships, that is, *C*_*m*_ and *C*_*d*_, respectively.

Various kinship structures and descent systems have evolved. Figure 2 shows some examples. In figure 2*a*, only one clan, namely A (yellow), exists and marriage occurs within it, representing clan endogamy. Here, traits and preferences do not diverge. In figure 2*b*, two clans, namely A (yellow) and B (green), prefer each other (A ⇔ B), representing dual organization. In this case, traits and preferences diverge in (*t*_2_, *p*_2_) space only. One can interpret this as a system in which maternally inherited traits *t*_2_ are solely referred to for marriage and descent. Hence, a matrilineal descent system evolves. In figure 2*c*, three clans, namely A (green), B (yellow) and C (orange), prefer other clans cyclically (A ⇒ B ⇒ C ⇒ A), representing generalized exchange. Here, traits and preferences diverge only in (*t*_1_, *p*_1_) space. One can interpret this as a system in which paternally inherited traits *t*_1_ are solely referred to for marriage and descent. Hence, a patrilineal descent system evolves. In figure 2*d*, four clans, namely, A_1_ (orange), A_2_ (pink), B_1_ (yellow), and B_2_ (green) exhibit pairwise mating preferences (A_1_ ⇔ B_2_ and A_2_ ⇔ B_1_) and descent relationships (A_1_ ↔ A_2_ and B_1_ ↔ B_2_). Specifically, restricted exchange has evolved. Here, both maternally and paternally inherited traits significantly diverge. Hence, a double descent system evolves.

**Figure 3.**
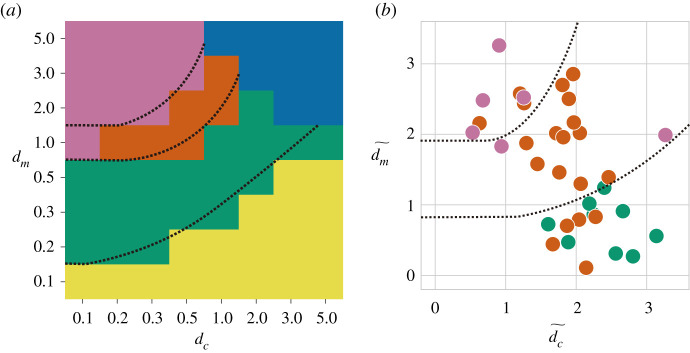
Phase diagrams on kinship structures. The figures show the classes of kinship structures that evolve the most frequently for each environmental parameter *d*_*c*_ and *d*_*m*_, both theoretically and empirically. Clan endogamy is plotted in yellow, dual organization in green, generalized exchange in orange and restricted exchange in pink. Conditions leading to the extinction of all societies are plotted in blue. The dashed lines represent the rough phase boundaries of the structures. Boundaries approach *d*_*m*_/*d*_*c*_ = *C* asymptotically when *d*_*c*_ is large and *d*_*m*_ = *C* ′ when *d*_*c*_ is small, according to the analytical calculations in the electronic supplementary material. (*a*) Theoretical phase diagram of kinship structures. We calculated the frequencies of each kinship structure using 100 trials for each *d*_*c*_ and *d*_*m*_ value. The figure illustrates the structure that evolved most frequently under each condition. Here, the number of societies in the system, *N*_*s*_ = *N*_*f*_ = 50, and *μ* = 0.1. (*b*) Empirical phase diagram of kinship structures, except for clan endogamy. By analysing the SCCS, we estimated the parameters for each society and plotted the dependencies of kinship structures on them. The estimated $\tilde{d_{c}}$ and $\tilde{d_{m}}$ are relative values, compared to *d*_*c*_ and *d*_*m*_. (See electronic supplementary material, figure S6 for the empirical phase diagram of kinship structures, including clan endogamy.) (Online version in colour.)

Indeed, various kinship structures and descent systems evolve even under the same environmental parameter values *d*_*c*_ and *d*_*m*_. However, statistically, evolved kinship structures and descent systems depend on parameter values. We conducted an evolutionary simulation 100 times for each condition and counted the frequencies with which each structure evolved. Figure 3*a* shows the parameter dependence of kinship structures as a phase diagram, by plotting the structure that evolved the most frequently in each condition. When *d*_*c*_ far exceeded *d*_*m*_, clan endogamy (yellow) evolved most frequently. As *d*_*m*_ increased relative to *d*_*c*_, the emergent structure changed to dual organization (green), generalized exchange (orange) and then to restricted exchange (pink). When both *d*_*c*_ and *d*_*m*_ are very large, all societies are extinct (blue). When *d*_*c*_ is small and *d*_*m*_ is large, societies can be composed of several endogamous clans as shown in electronic supplementary material, figure S2. However, this rarely occurs within the current parameter regions. Note that the diagram is qualitatively robust to the choice of initial conditions.

These successive transitions were accompanied by an increased number of clans within society and a decreased probability of sustaining structures against population fluctuations. To estimate the phase boundary, we analytically calculated conditions for each structure to evolve. We explain it below briefly (see the electronic supplementary material for further details). We assume that the centres of the groom and bride clans deviate with the order of the mutation rate *μ* due to fluctuations. Because of this deviation, the degree of cooperation of the mate is reduced by the factor exp(−*αμ*^2^) from that of the kin (where α∼O(1)). For example, in clan endogamy, every family is kin and rival simultaneously, whereas in dual organization, a half is kin and rival, and the other half is mate. Then, recalling the above reduction, the conditions in which dual organization is more adaptive than clan endogamy are given by

3.1 $$p_{C}\exp(-d_{c}\cdot 0-d_{m}\cdot 1)$$

3.2 $$\;<p_{D}\exp\left(-d_{c}\left(\frac{1}{2}-\frac{1}{2}\exp(-\alpha \mu^{2})\right)-d_{m}\cdot \frac{1}{2}\right),$$

3.3 $$\Leftrightarrow \frac{d_{m}}{d_{c}}>1-\exp(-\alpha \mu^{2})+\frac{2}{d_{c}}\log\frac{\;p_{C}}{p_{D}},$$

where *p*_*C*_ and *p*_*D*_ denote the sustenance probability for clan endogamy and dual organization, respectively (see electronic supplementary material for their estimation). The transition to generalized or restricted exchange is estimated similarly. In short, the transitions occur if the pressure for segmentation caused by large *d*_*m*_ values exceeds that for clustering by *d*_*c*_ and the relative probability for sustaining structures. Then, we derived the phase boundaries of *d*_*m*_/*d*_*c*_ = *C* asymptotically when *d*_*c*_ was large and *d*_*m*_ = *C*′ when *d*_*c*_ was small, as shown in figure 3.

**Figure 4.**
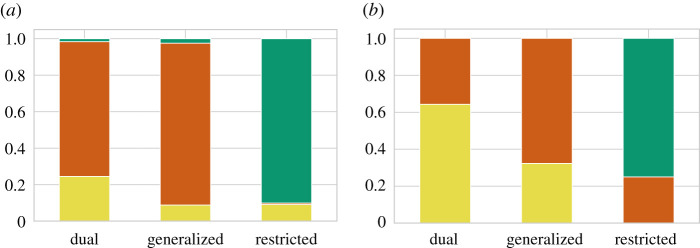
Frequency of descent systems for each kinship structure. Figures show the frequency of each descent system, both theoretically and empirically. The frequencies of matrilineal (yellow), patrilineal (orange) and double descent (green) systems are shown. (*a*) Theoretical phase diagram. The model was simulated by changing the *d*_*c*_ and *d*_*m*_ values. We calculated the frequencies of each descent system for each kinship structure. Here, *N*_*s*_ = 50, *N*_*f*_ = 30, and *μ* = 0.1. (*b*) Empirical phase diagram. By analysing the SCCS, we identified the descent systems and kinship structures of each society. We counted the frequencies of each descent system for each kinship structure. (Online version in colour.)

Figure 4*a* shows the dependency of descent systems on kinship structures. Double descent is the most frequent in restricted exchange. Patrilineal descent is the most frequent in dual organization and generalized exchange, whereas matrilineal descent is more frequent in dual organization.

Electronic supplementary material, figure S3 shows the dependence of phase diagrams of kinship structures on the number of societies in the system *N*_*s*_, the number of families within a society *N*_*f*_, and the mutation rate *μ*. As *N*_*s*_ increases, the group-level selective pressure is strong and restricted exchange evolves across broader parameter regions, whereas the region of dual organization narrows. Larger *N*_*f*_ results in smaller fluctuations in the population of clans and thus generalized and restricted exchanges evolve across broader parameter regions. As *μ* increases, restricted or generalized exchanges disappear due to larger fluctuations in traits and preferences.

## 4. Empirical data analyses

We then tested our results on the phase diagrams of kinship structure and descent system using the SCCS database [17,31]. We classified the kinship structures of each society by identifying the composition of clans, and marriage and descent rules between them. See electronic supplementary material, table S2 for further details. Of the 186 societies in SCCS, we identified 87 as clan endogamy, 14 as dual organization, 33 as generalized exchange and 12 as restricted exchange. Forty societies were excluded from the analyses because their marriage rules prohibit within-clan (or family) marriage only and do not specify any preferable mates. (Note that, in this paper, we focus on the system in which families choose a mate from a specific clan.) Electronic supplementary material, figures S4 and S5 show the geographic distributions of kinship structures and descent systems, respectively. Each structure is distributed globally, without a clear spatial pattern, suggesting that kinship structures in each region were achieved by cultural adaptation, rather than cultural transmission; this must be further investigated by phylogenetic comparative analysis.

Next, we conducted Spearman’s rank correlation analyses and calculated the correlation between SCCS variables and kinship structures. The database contains various variables of socio-ecological factors. Although there are no variables in SCCS that exactly correspond to *d*_*c*_ and *d*_*m*_, *d*_*c*_ can be related to the extent of social unity and external warfare, and *d*_*m*_ to the attitude towards adultery and the extent of internal warfare. Notably, marriage conflict over mates arises at the family or kin group level, whereas inter-society conflict requires cooperation across different kin groups. Thus, violence within a society is related to *d*_*m*_, and that involving other societies to *d*_*c*_. We calculated the correlation for each variable and listed the variables in descending order in the absolute value of the correlation. We then found that the variables related to *d*_*c*_ and *d*_*m*_ were located at the top of the list (rather than the middle or bottom). The variables that were highly correlated with kinship structures are listed in electronic supplementary material, table S3. Among them, we show the variables that can be related to *d*_*c*_ and *d*_*m*_ in table 2.

**Table 2.** Correlations between SCCS variables and kinship structures (excerpt). For pairs of kinship structures, the Spearman’s rank correlation between the SCCS variables and the structures was calculated. Then, the absolute values of the correlation were averaged for each pair. We list the variables that exhibited high correlations and were relevant to *d*_*c*_ and *d*_*m*_, along with the average value of the correlation (corr.) and the corresponding parameters in the model. See electronic supplementary material, table S3 for further information.

variable	corr.	model
tributary payments or taxation	0.58	*d*_*c*_
violence against other ethnic groups	0.57	*d*_*c*_
external warfare	0.54	*d*_*c*_
hostility towards other ethnic groups	0.48	*d*_*c*_
cross-cutting ties	0.45	*d*_*c*_
conflict within the society	0.59	*d*_*m*_
violence within the Society	0.55	*d*_*m*_
disapproval of rape	0.53	*d*_*m*_
disapproval of premarital sex	0.51	*d*_*m*_
disapproval of incest	0.41	*d*_*m*_

We estimated *d*_*c*_ using the variables pertaining to social unity (*tributary payments or taxation* and *cross-cutting ties*) and society-level conflict that requires immense cooperation within society (*violence against other ethnic groups*, *external warfare* and *hostility towards other ethnic groups*). We estimated *d*_*m*_ using the variables pertaining to attitudes towards adultery (*disapproval of rape*, *disapproval of premarital sex* and *disapproval of incest*) and intra-society conflict (*conflict within the society* and *violence within the society*).

Next, we normalized the values of each variable to set the mean to 0 and variance to 1. We changed the sign if necessary so that larger values corresponded to larger *d*_*c*_ or *d*_*m*_. For some societies, the data for some variables were lacking; however, we averaged the available values to estimate $\tilde{d_{c}}$ and $\tilde{d_{m}}$ (hereafter, values with tilde represent those estimated by empirical data analyses). We added a constant to set the minimum values of $\tilde{d_{c}}$ and $\tilde{d_{m}}$ to 0, because *d*_*c*_ and *d*_*m*_ were positive values in our model. Although the absolute magnitudes were not comparable, $\tilde{d_{c}}$ and $\tilde{d_{m}}$ would be positively correlated with *d*_*c*_ and *d*_*m*_, respectively. The empirical dependence of the kinship structures on $\tilde{d_{c}}$ and $\tilde{d_{m}}$ is shown in figure 3*b*. The results were qualitatively consistent with the theoretical phase diagrams for *d*_*c*_ and *d*_*m*_. As $\tilde{d_{m}}/\tilde{d_{c}}$ increased, kinship structures changed from dual organization to generalized exchange, and then to restricted exchange. The consistency between data and model results was worse for clan endogamy, as observed in electronic supplementary material, figure S6. This may be because real societies with such structures can have social systems other than kinship, regulating social unity and suppressing marital competition.

The frequency of each descent system in each kinship structure was also calculated and shown in figure 4*b*. Patrilineal descent is the most frequent in generalized exchange. The frequency of matrilineal descent was larger in dual organization compared to that in generalized exchange. These are comparable with the model results, although the correspondence was much weaker than that of the kinship structures.

## 5. Discussion

By considering cooperation among kin and mates, as well as competition among rivals, we theoretically demonstrated that families formed some clusters in traits and preferences. Families within a cluster are recognized as cultural kin, and marriage occurs only among families from different clusters. Hence, the clusters of families that emerged in our model can be interpreted as clans. Initially, uniform traits are discretized into several clusters, i.e. clans. Furthermore, by tracing marriage and descent relationships between clans, the evolution of various kinship structures was observed. The traits and preferences were differentiated involving either paternally or maternally inherited ones only, or both. This demonstrates the evolution of patrilineal, matrilineal and double descent systems, respectively. Additionally, we revealed that the parameters related to *d*_*c*_ and *d*_*m*_ in our model can be considered as significant explanatory variables for different kinship structures, by analysing the ethnographic data of 146 societies. By estimating *d*_*c*_ and *d*_*m*_ from the data, we demonstrated consistency between the theoretical and empirical results of the parameter dependencies of the kinship structures and descent systems.

In cultural anthropology, ‘descent theory’ and ‘alliance theory’ have been proposed to explain kinship structures. They emphasize cooperation fostered by shared descent and marriage, respectively [1,44]. Here, we added the effect of marital competition. By introducing the evolutionary pressure to increase cooperation among kin and mates, and to decrease competition among rivals, we illustrated that diverse kinship structures evolve depending on the pressures. Generally, it is difficult to compare the historical consequences of the formation of kinship structures since chronological records are rarely available. Nevertheless, we can explain how each structure was sustained for a specific condition. Indeed, Lévi–Strauss demonstrated several examples of the sustenance of kinship structures. Cultural groups become divergent owing to population growth and internal conflict. Simultaneously, however, they are united by marital relationships. Even if some of the population is damaged, the structures eventually recover within several generations [1]. Furthermore, we can compare our theoretical results with empirical data, and their consistency supports the plausibility of our scenario.

According to the simulations, kinship structures evolve depending on the two pressures parametrized by *d*_*c*_ and *d*_*m*_. That is, the importance of cooperating among kin and mates and that of avoiding marital competition, determined by environmental conditions. For example, *d*_*c*_ is related to the frequency and importance of public works or massive violence in societies, whereas *d*_*m*_ is related to the scarcity of mates. When the pressure for cooperation is much larger than the avoidance of competition, societies comprise one or several clans and most families are united as kin or mates. By contrast, as the importance of avoiding competition increases, dividing societies into more clans becomes more adaptive. Hence, as *d*_*m*_/*d*_*c*_ increases, the emergent structures change from clan endogamy to dual organization, generalized exchange, and then to restricted exchange. In cultural anthropology, dual organization is categorized as the simplest form of restricted exchange, by focusing on *C*_*m*_ = 2 [1]. Our results, however, suggest that it is closer to generalized exchange concerning environmental dependencies as expected by focusing on *C*_*d*_ = 1.

Furthermore, diverse descent systems evolved in our model. Under the moderate values of *d*_*m*_, either paternally or maternally inherited traits and preferences solely diverge because of the symmetry breaking. This leads to patrilineal or matrilineal descent, respectively. In our simulation, patrilineal descent evolved more frequently than did matrilineal. As mentioned above, the sole asymmetry of sex lies in the process of choosing a mate; that is, women (or their families) prefer certain men’s traits. Thus, the selection pressure to favour those men with preferable traits leads to the divergence of paternally inherited traits. In real-world data too, patrilineal descent is more frequent than matrilineal descent. However, in our model, the frequency of patrilineal descent was too large. In some societies, grooms’ families choose brides. Furthermore, other aspects cannot be neglected. For example, with paternal uncertainty, matrilineal descent will likely evolve [19]. The necessity for cooperation generally differs for men and women, depending on subsistence patterns or frequency of warfare [2]. For further discussion on the evolution of descent systems, these biases should be considered. Nonetheless, our study shows the emergence of significant traits that are frequently inherited paternally.

In the empirical data analysis, we found the correlation between kinship structures and the status of wives, as well as *d*_*c*_ and *d*_*m*_ (see electronic supplementary material, table S3). Specifically, gender inequality concerning the wives’ status increased in the following order: restricted exchange, dual organization and generalized exchange (although this cannot be directly related to the gender balance in general). Thus, in this aspect, it may be reasonable to assume that dual organization is more similar to restricted exchange than to generalized exchange. As the empirical data and our model exhibit, the descent system is more biased towards patrilineal descent in generalized exchange and less so in dual organization, whereas double descent is adopted in restricted exchange. In societies with patrilineal descent systems, wives join husbands’ groups after marriage [2]. If male dominance is more frequently observed therein, we can explain the above trend. Generalized exchange exhibits the largest inequality, whereas it is between restricted exchange and dual organization regarding environmental dependence. This suggests the benefit of analysing kinship structures to elucidate other cultural aspects of society.

Apart from the parameters *d*_*c*_ and *d*_*m*_, the mutation rate *μ*, the number of competing societies *N*_*s*_ and the initial number of families within a society *N*_*f*_ are also relevant in determining kinship structures (see electronic supplementary material, figure S3 and table S3). Electronic supplementary material, figure S3 as well as our analytical calculations suggest that sophisticated structures, such as generalized and restricted exchanges, are more fragile due to the larger fluctuation under smaller *N*_*s*_ or *N*_*f*_, or larger *μ*. Electronic supplementary material, table S3 suggests that such sophisticated structures are correlated with large *N*_*s*_ and small *N*_*f*_. Hence, the theoretical trend was empirically verified for *N*_*s*_, but not for *N*_*f*_. In reality, if *N*_*f*_ is larger, clan endogamy is the most frequent. This may be due to the development of social organizations other than kinship, such as polities, which would regulate the relationships of people in larger societies [2]. The emergence of such organizations is also a cultural evolutionary phenomenon; however, it is beyond the scope of our model. Regarding *μ*, it will be determined by how traits and preferences are inherited, and social norms regulate precise inheritance [22]. Thus far, however, its estimation from the data remains a task for the future.

Our model shares some similarities with the biological model for sympatric speciation. The evolution of several endogamous groups is shown as a result of evolved mating preferences [45] or resource competition [46]. Conversely, humans develop the ability to recognize kin, leading to organizing the affinal network of groups by exogamy [47,48]. Our model includes cooperation among mates and thus, exhibits the emergence of diverse kinship structures more than the mere divergence of groups.

The present study has some limitations. In the model, we only focused on societies in which marriage and descent rules were strictly determined by customs. Additionally, our model concerns the elementary structures of kinship, where paternal and maternal traits are referred to independently [1]. In real societies, as population size expands, the unity of kin groups weakens and marriage rules are relaxed, such that only marriage within the clan or nuclear family is prohibited [49]. Such rules to exclude unpreferable mates cannot evolve in our model. To cover the observed rules comprehensively, a new model needs to be developed to consider positive and negative preferences for mates. The descent rules also change, such that they can refer either to the father or the mother in each generation by choice, or genealogical distance only. This occurs in complex structures of kinship [17,31].

Moreover, we could only analyse the correlations between ethnographic variables and kinship structures. We could not assign the variables to *d*_*c*_ and *d*_*m*_ *a priori*. Therefore, our estimation of $\tilde{d_{c}}$ and $\tilde{d_{m}}$ may seem arbitrary. To measure these variables directly, it is thus necessary to collaborate with field studies. It is also desirable to conduct further analyses, such as classification learning; however, it was unfeasible in the current study owing to data insufficiency. Phylogenetic comparative analysis is also necessary to control statistical non-independence owing to shared ancestry [25]. Furthermore, because of the lack of chronological data, we could not analyse the causal relationships between social structures and cultural conditions related to *d*_*c*_, *d*_*m*_ and other parameters.

Social structures, such as kinship, are formed through interactions among people over many generations. In this paper, we theoretically demonstrate such formation of macroscopic social structures through microscopic family behaviours. It is considered difficult to explain such complex systems from basic conditions solely using simple correlation analyses [36]. Combined with theoretical simulations of a simple constructive model and empirical data analyses, we have explained the dependence of various kinship structures around the world upon the degrees of cooperation and avoidance of competition. Theoretical studies, as shown here, produce explanatory scenarios by referring to empirical studies and propose relevant variables to be measured in the field. Empirical studies in the field describe notable anthropological phenomena and enable the measurement of variables to test theories. Such collaboration of theoretical and empirical studies will contribute to discussing the emergence of complex social structures and unveiling universal features in anthropology.

## 6. Methods

### (a) Algorithm

To simulate population growth considering social interactions of families, the degrees of cooperation and competition were calculated by comparing trait and preference values with a tolerance parameter *τ*. Hence, families *i* and *j* cooperate if |***t***^*i*^− ***t***^*j*^|/*τ*, |***t***^*i*^− ***p***^*j*^|/*τ* or |***p***^*i*^− ***t***^*j*^|/*τ* is sufficiently small. These conditions correspond to *i* and *j* being cultural kin, the women in *j* preferring men in *i* and the women in *i* preferring men in *j*, respectively. Families *i* and *j* compete if they prefer similar families, that is, if |***p***^*i*^− ***p***^*j*^|/*τ* is sufficiently small. Then, the possibility of marriage and the degrees of cooperation and competition were measured using a Gaussian function. For example, the degree of cooperation between cultural kin is given by exp(−|***t***^*i*^− ***t***^*j*^|^2^/*τ*^2^).

We adopted the following algorithm for population changes in families: For family *i* and time step *n*, the numbers of unmarried men and unmarried women are denoted by *M*^*i*^(*n*) and *F*^*i*^(*n*), respectively. The probability for family *i* to offer family *i′* for marriage is denoted by *P(i′).* The intrinsic growth rate is denoted by *b*. We represented the set of families in society as Φ, the families that accept men in the family *i* as grooms as *i*′, and the children's families of men in family *i* and women in family *i*' as i*. The population change in family *i* is given by

6.1 $$d_{i,j}=\min(|t^{i}-t^{j}|,|p^{i}-t^{j}|,|t^{i}-p^{j}|),$$

6.2 $${\text{friend}}^{i}(n)=\sum_{\;j\in \Phi }\frac{\exp(-d_{i,j}^{2}/\tau^{2})}{\#\Phi },$$

6.3 $${\text{rival}}^{i}(n)=\sum_{\;j\in \Phi }\frac{\exp(-|p^{i}(n)-p^{j}(n){|}^{2}/\tau^{2})}{\#\Phi },$$

6.4 $$r=b\exp(-d_{c}(1-{\text{friend}}^{i}\;(n))-d_{m}\;{\text{rival}}^{i}(n)),$$

6.5 $$M^{i}(n)=\text{Poisson}(r),\;F^{i}(n)=\text{Poisson}(r),$$

6.6 $$P(i^{\prime })=\frac{\exp(-|t^{i}(n)-p^{i^{\prime }}(n){|}^{2}/\tau^{2})}{\sum_{\;j\in \Phi }\exp(-|t^{i}(n)-p^{j}(n){|}^{2}/\tau^{2})},$$

and

6.7 $$t^{i^{*}}(n+1)=(t_{1}^{i},t_{2}^{i^{\prime }})+\eta ,\;p^{i^{*}}(n+1)=(p_{1}^{i},p_{2}^{i^{\prime }})+\eta .$$

The population growth of each family depends on *friend* and *rival*, as given by equations (6.2) and (6.3), respectively. The number of unmarried children in each family follows a Poisson distribution, as given by equations (6.4) and (6.5), respectively. People are married according to the traits and preferences of their families, as shown in equation (6.6). After marriage, couples give birth to children and die. At this time, children inherit the traits and preferences of parents by adding the noise component ***η*** to them. This comprises two independent normal variates with mean 0 and variance *μ*^2^, as shown in equation (6.7). Unmarried people can join the mating in the next step. However, those who cannot find mates within two steps die without having children. Here, we assumed monogamy; however, the result was qualitatively independent of such a marriage system.

## Data accessibility

Source codes for the model can be found here: https://github.com/KenjiItao/clan.git. The data are provided in electronic supplementary material [50].

## References

[1] Lévi-Strauss C. 1949 Les structures élémentaires de la parenté. Paris, France: Presses Universitaires de France.

[2] Service E. 1962 Primitive social organization: an evolutionary perspective. Random House Studies in Anthropology. New York, NY: Random House.

[3] Fox R. 1983 Kinship and marriage: an anthropological perspective, *vol. 50*. Cambridge, UK: Cambridge University Press.

[4] Maddock K. 1969 Alliance and entailment in Australian marriage. Aust. J. Anthropol. **7**, 19-26. (10.1111/j.1835-9310.1969.tb00382.x)

[5] Leach ER. 1954 Political systems of highland Burma. London, UK: Bell & Sons.

[6] Malinowski B. 1963 Sex, culture, and myth. London, UK: R. Hart-Davis.

[7] Murdock GP. 1949 Social structure. New York, NY: Macmillan.

[8] Hopkins K. 1980 Brother--sister marriage in roman Egypt. Comp. Stud. Soc. Hist. **22**, 303-354. (10.1017/S0010417500009385)

[9] Hill KR et al. 2011 Co-residence patterns in hunter-gatherer societies show unique human social structure. Science **331**, 1286-1289. (10.1126/science.1199071) 21393537

[10] Shenk MK, Mattison SM. 2011 The rebirth of kinship. Hum. Nat. **22**, 1-15. (10.1007/s12110-011-9105-9) 22388799

[11] Passmore S, Barth W, Quinn K, Greenhill SJ, Evans N, Jordan FM. 2021 Kin against kin: internal co-selection and the coherence of kinship typologies. Biol. Theory **16**, 1-18. (10.1007/s13752-021-00379-6)

[12] White HC. 1963 An anatomy of kinship: mathematical models for structures of cumulated roles. Englewood Cliffs, NJ: Prentice-Hall.

[13] Romney AK, Epling PJ. 1958 A simplified model of kariera kinship. Am. Anthropol. **60**, 59-74. (10.1525/aa.1958.60.1.02a00070)

[14] Lévi-Strauss C. 1965 The future of kinship studies. The Huxley Memorial Lecture 1965. *Proc. of the Royal Anthropological Institute of Great Britain and Ireland*, *1965*, pp. 13–22. (10.2307/3031752)

[15] Murdock GP. 1940 Double descent. Am. Anthropol. **42**, 555-561. (10.1525/aa.1940.42.4.02a00020)

[16] Goody J. 1961 The classification of double descent systems. Curr. Anthropol. **2**, 3-25. (10.1086/200156)

[17] Murdock GP, White DR. 1969 Standard cross-cultural sample. Ethnology **8**, 329-369. (10.2307/3772907)

[18] Hartung J. 1981 Paternity and inheritance of wealth. Nature **291**, 652-654. (10.1038/291652a0)

[19] Holden CJ, Sear R, Mace R. 2003 Matriliny as daughter-biased investment. Evol. Hum. Behav. **24**, 99-112. (10.1016/S1090-5138(02)00122-8)

[20] Alvard MS. 2003 Kinship, lineage, and an evolutionary perspective on cooperative hunting groups in Indonesia. Hum. Nat. **14**, 129-163. (10.1007/s12110-003-1001-5) 26190056

[21] Alvard M. 2011 Genetic and cultural kinship among the lamaleran whale hunters. Hum. Nat. **22**, 89-107. (10.1007/s12110-011-9104-x) 22388802

[22] Cavalli-Sforza LL, Feldman MW. 1981 Cultural transmission and evolution: a quantitative approach. Princeton, NJ: Princeton University Press. 7300842

[23] Guglielmino CR, Viganotti C, Hewlett B, Cavalli-Sforza LL. 1995 Cultural variation in Africa: role of mechanisms of transmission and adaptation. Proc. Natl Acad. Sci. USA **92**, 7585-7589. (10.1073/pnas.92.16.7585) 11607569 PMC41384

[24] Mulder MB, George-Cramer M, Eshleman J, Ortolani A. 2001 A study of east African kinship and marriage using a phylogenetically based comparative method. Am. Anthropol. **103**, 1059-1082. (10.1525/aa.2001.103.4.1059)

[25] Minocher R, Duda P, Jaeggi AV. 2019 Explaining marriage patterns in a globally representative sample through socio-ecology and population history: a Bayesian phylogenetic analysis using a new supertree. Evol. Hum. Behav. **40**, 176-187. (10.1016/j.evolhumbehav.2018.11.003)

[26] Traulsen A, Nowak MA. 2006 Evolution of cooperation by multilevel selection. Proc. Natl Acad. Sci. USA **103**, 10 952-10 955. (10.1073/pnas.0602530103) 16829575 PMC1544155

[27] Takeuchi N, Hogeweg P, Kaneko K. 2017 The origin of a primordial genome through spontaneous symmetry breaking. Nat. Commun. **8**, 1-11. (10.1038/s41467-017-00243-x) 28811464 PMC5557888

[28] Spencer CS, Redmond EM. 2001 Multilevel selection and political evolution in the valley of Oaxaca, 500–100 BC. J. Anthropol. Archaeol. **20**, 195-229. (10.1006/jaar.2000.0371)

[29] Turchin P, Gavrilets S. 2009 Evolution of complex hierarchical societies. Soc. Evol. Hist. **8**, 167-198.

[30] Itao K, Kaneko K. 2020 Evolution of kinship structures driven by marriage tie and competition. Proc. Natl Acad. Sci. USA **117**, 2378-2384. (10.1073/pnas.1917716117) 31964846 PMC7007516

[31] Kirby KR et al. 2016 D-place: a global database of cultural, linguistic and environmental diversity. PLoS ONE **11**, e0158391. (10.1371/journal.pone.0158391) 27391016 PMC4938595

[32] Marsh RM. 1967 Comparative sociology. New York, NY: Harcourt, Brace & World.

[33] Bernard HR. 2017 Research methods in anthropology: qualitative and quantitative approaches. New York, NY: Rowman & Littlefield.

[34] Hoben AD, Buunk AP, Fisher ML. 2016 Factors influencing the allowance of cousin marriages in the standard cross cultural sample. Evol. Behav. Sci. **10**, 98-108. (10.1037/ebs0000034)

[35] White DR, Burton ML. 1988 Causes of polygyny: ecology, economy, kinship, and warfare. Am. Anthropol. **90**, 871-887. (10.1525/aa.1988.90.4.02a00060)

[36] Rácz P, Passmore S, Jordan FM. 2020 Social practice and shared history, not social scale, structure cross-cultural complexity in kinship systems. Top. Cogn. Sci. **12**, 744-765. (10.1111/tops.12430) 31165555 PMC7318210

[37] Itao K, Kaneko K. 2021 Evolution of family systems and resultant socio-economic structures. Humanit. Soc. Sci. Commun. **8**, 1-11. (10.1057/s41599-021-00919-2)

[38] Traulsen A, Nowak MA. 2006 Evolution of cooperation by multilevel selection. Proc. Natl Acad. Sci. USA **103**, 10 952-10 955. (10.1073/pnas.0602530103) 16829575 PMC1544155

[39] Wilson DS, Wilson EO. 2003 The quarterly review of biology. Lancet **207**, 918. (10.1016/S0140-6736(00)93717-6)

[40] Lévi-Strauss C et al. 1962 La pensée sauvage, *vol. 289*. Paris, France: Plon.

[41] Sperber D, Hirschfeld LA. 2004 The cognitive foundations of cultural stability and diversity. Trends Cogn. Sci. **8**, 40-46. (10.1016/j.tics.2003.11.002) 14697402

[42] Chagnon NA. 1988 Life histories, in blood a tribal revenge, and population warfare. Science **239**, 985-992. (10.1126/science.239.4843.985) 17815700

[43] Pelleg D, Moore AW. 2000 *X*-means: extending *k*-means with efficient estimation of the number of clusters. In *Proc. of the Seventeenth Int. Conf. on Machine Learning* (ed. P Langley), pp. 727–734. San Francisco, CA: Morgan Kaufmann Publishers Inc.

[44] Leach ER. 1982 Social anthropology. London, UK: William Collins Sons & Co. Ltd.

[45] Dieckmann U, Doebeli M. 1999 On the origin of species by sympatric speciation. Nature **400**, 354-357. (10.1038/22521) 10432112

[46] Kaneko K, Yomo T. 2000 Sympatric speciation: compliance with phenotype diversification from a single genotype. Proc. R. Soc. Lond. B **267**, 2367-2373. (10.1098/rspb.2000.1293) PMC1690829 11133025

[47] Chapais B. 2009 Primeval kinship: how pair-bonding gave birth to human society. Cambridge, MA: Harvard University Press.

[48] Planer RJ. 2020 Towards an evolutionary account of human kinship systems. Biol. Theory **16**, 1-14. (10.1007/s13752-019-00339-1)

[49] Harrell S. 1997 Human families. Boulder, CO: Westview Press.

[50] Itao K, Kaneko K. 2022 Emergence of kinship structures and descent systems: multi-level evolutionary simulation and empirical data analysis. *Figshare*. 10.1098/rspb.2021.2641 PMC8864366 35193405

