# Supplementary material for: Emergence of kinship structures and descent systems: multi-level evolutionary simulation and empirical data analysis
Source: Proc Biol Sci. 2022 Feb 23;289(1969):20212641. doi: 10.1098/rspb.2021.2641 (PMC8864366; doi:10.1098/rspb.2021.2641)
Supplement: Supplementary file 1 [file rspb20212641_review_history.pdf]

THE ROYAL SOCIETY
PUBLISHING

PROCEEDINGS B

Emergence of kinship structures and descent systems: multi-level evolutionary simulation and empirical data analysis

Kenji Itao and Kunihiko Kaneko

Article citation details
Proc. R. Soc. B **289**: 20212641.
http://dx.doi.org/10.1098/rspb.2021.2641

Review timeline

Original submission:	18 May 2021
1st revised submission:	5 December 2021
2nd revised submission:	24 January 2022
Final acceptance:	25 January 2022

Note: Reports are unedited and appear as submitted by the referee. The review history appears in chronological order.

Review History

RSPB-2021-1136.R0 (Original submission)

Review form: Reviewer 1

Recommendation
Major revision is needed (please make suggestions in comments)

Scientific importance: Is the manuscript an original and important contribution to its field?
Good

General interest: Is the paper of sufficient general interest?
Good

Quality of the paper: Is the overall quality of the paper suitable?
Poor

Is the length of the paper justified?
Yes

Should the paper be seen by a specialist statistical reviewer?
No

Reports © 2022 The Reviewers; Decision Letters © 2022 The Reviewers and Editors; Responses © 2022 The Reviewers, Editors and Authors. Published by the Royal Society under the terms of the Creative Commons Attribution License http://creativecommons.org/licenses/by/4.0/, which permits unrestricted use, provided the original author and source are credited

Do you have any concerns about statistical analyses in this paper? If so, please specify them explicitly in your report.
No

It is a condition of publication that authors make their supporting data, code and materials available - either as supplementary material or hosted in an external repository. Please rate, if applicable, the supporting data on the following criteria.

Is it accessible?
No

Is it clear?
No

Is it adequate?
No

Do you have any ethical concerns with this paper?
No

Comments to the Author
This paper addresses an interesting and important topic, but it is extremely hard to understand. This is especially a problem in simulation studies because the causal machinery is not so easy to see. Here even the most basic assumptions are hard to figure out, so I recommend the authors be asked to undertake a major revision with the goal of explicating the structure of their model and providing intuitions for the basic results.

Here is a list of some of the things that need to be clarified:

1. Is their migration between groups? Migration is crucial for the maintenance of variation among groups which is in turn crucial for multi-level selection. When traits are discrete, the rare type disadvantage selection assume will act to increase variation among groups. But in the model behavioral and preference traits vary continuously, and this means that even a small amount of migration will homogenize groups. This is crucial, I think, but I do not find any mention of migration in the text.

2. The relationship between the preference structures specified by the inherited traits and the marriage rules is not clear. The results are not so interesting if the marriage rules (not preferences) are imposed exogenously, but I can't figure out exactly what is going on.

3. A crucial result is that evolution creates preference structure so that women in one family prefer the trait (t value) in other families. This seems to be the outcome of simulations, but the paper gives no intuition for why this should be the case.

4. Much data suggests that parents are relatively unimportant in cultural transmission, so the assumption of pure vertical transmission should be tested. This could strengthen the model because if kids learn from other same sex family members, this will create the cultural analog of inbreeding (e.g. you learn from dad and dad's relatives who learned from culturally related members of the previous generation). This will tend to increase between family variation all other things being equal.

Review form: Reviewer 2 (Robert Gilman)

Recommendation
Accept with minor revision (please list in comments)

Scientific importance: Is the manuscript an original and important contribution to its field?
Excellent

General interest: Is the paper of sufficient general interest?
Excellent

Quality of the paper: Is the overall quality of the paper suitable?
Excellent

Is the length of the paper justified?
Yes

Should the paper be seen by a specialist statistical reviewer?
No

Do you have any concerns about statistical analyses in this paper? If so, please specify them explicitly in your report.
No

It is a condition of publication that authors make their supporting data, code and materials available - either as supplementary material or hosted in an external repository. Please rate, if applicable, the supporting data on the following criteria.

> **Is it accessible?**
> Yes
>
> **Is it clear?**
> Yes
>
> **Is it adequate?**
> Yes

Do you have any ethical concerns with this paper?
No

Comments to the Author
I have uploaded my review as a PDF to preserve small bits of notation. (See Appendix A)

Review form: Reviewer 3

Recommendation
Reject – article is not of sufficient interest (we will consider a transfer to another journal)

Scientific importance: Is the manuscript an original and important contribution to its field?
Acceptable

General interest: Is the paper of sufficient general interest?
Marginal

Quality of the paper: Is the overall quality of the paper suitable?
Acceptable

Is the length of the paper justified?
No

Should the paper be seen by a specialist statistical reviewer?
Yes

Do you have any concerns about statistical analyses in this paper? If so, please specify them explicitly in your report.
Yes

It is a condition of publication that authors make their supporting data, code and materials available - either as supplementary material or hosted in an external repository. Please rate, if applicable, the supporting data on the following criteria.

Is it accessible?
Yes

Is it clear?
Yes

Is it adequate?
Yes

Do you have any ethical concerns with this paper?
No

Comments to the Author
My thanks to the authors and editors for this patience on a review for this paper.

As an evolutionary anthropologist interested in the evolution of human kinship I was pleased to be offered this paper to review. I think there are some excellent ideas here and I am delighted to see the use of ABMs alongside the empirical ethnographic data. However, I do not think it is suitable for PRSB for a few reasons I will explain briefly below.

The paper explores if various social, cultural and family-level parameters can produce kinship structures akin to the categories proposed by Levi-Strauss's alliance models. While very interesting and important to human kinship specialists, the paper is not motivated to be of interest to a wide evolution/biology/behaviour audience. Kinship is a complex topic in anthropology, and to be of broad interest beyond anthropologists the authors needed to make clear how understanding L-S's structures might shed more general light on human sociality, acting and cooperation. The structures themselves are taken-for-granted, and while they have been influential in anthropology since L-S's work in the 1950s, they are but one of a range of ways to categorise kinship arrangements in human societies. For a PRSB audience, we need a theoretical account of social behaviours grounded in evolutionary principles, and while the parameters in the ABM model could definitely be seen in that way the manuscript does not make the motivation for the model clear.

Despite very much being invested in understanding the work presented, I am sorry to say that I found this paper very hard to comprehend. From the outset it was not clear whether the existence of clans, or the existence of alliance structures, was the target of the model. When I tried to work my way through the specifics of the model I couldn't understand why there was multi-level population structure (families and societies) but no kinship organisation. There is strong evidence

from our primate cousins that early hominids would have had some form of kinship structure (see work by e.g. Silk 2021 or Chapais 2008). Comparative cross-cultural work shows us that foraging societies, to the extent that they can provide a picture of the range of pre-Neolithic social structures, have flexibility built into their structures. But I wasn't sure what extent of human history the modelling was meant to provide an explanation for - the original emergence of alliance structures, or their repeated change over cultural evolutionary time? This last point speaks to a wider issue, which is that the manuscript does not have any ethnographic examples to illustrate the phenomena being explained. To speak to an audience beyond modellers, I think this is quite crucial.

I reviewed from the perspective of a cross-cultural evolutionary anthropologist who studies kinship. I am familiar with and can follow ABMs, but I cannot attest to the rigour or quality of the simulation models and analytical approaches presented here -- they might be exemplary but it would be remiss of me to claim I can judge their accuracy. One point of particular difficulty for me was the "cultural traits" that were crucial to the ABM. Families had these traits, and had preferences for them. Some examples were given -- surnames, totems, and occupations -- all of which are phenomena which may denote clan membership in some societies, and this felt circular. I was very unclear on whether these traits could be any kind of shared public signifier, and whether they could only be symbolic. Occupation, for example, signals information about resources as well as social desirability.

I do think there is too much packed into this paper. It is the kind of work that would be better in another venue as a two-part paper Part 1: Model - Part 2: Empirical analyses, with ample space given to motivate the investigations and to describe and justify the parameters under study. The authors have made a valiant attempt at compressing a lot of work into a PRSB format but it is at the expense of clarity.

Finally, I felt on more familiar terrain with the final cross-cultural analyses in the paper, and I offer some specific remarks here which I hope the authors can use to improve their study.

* The section "Classification of Kinship Structures" in the Methods could be much clearer. Interpreting the SM tables and values of variables requires deep specialist knowledge of kinship jargon and worked ethnographic examples would be more tractable.

* Only 60/186 societies were included, because of the exclusive definitions of the four structures. The 186 societies in the SCCS are, to a first approximation, the most ethnographically well-described communities that anthropologists have studies, but L-S's framework can only account for kinship practices in a third of them. This weakens any claim of "universal anthropology" (the authors' choice of keywords).

* Because only 60 were identified, I was less convinced by the "matching" data presented in Figure 4. This is not only because of the small sample size, but because of phylogenetic and spatial autocorrelation effects. These still exist in the SCCS, despite wishful-thinking claims to the contrary.

* The authors then looked for explanatory variables for the kinship structures (line 340): "we conducted Spearman's rank correlations 340 between the SCCS variables and the structures." I think what they have done here is to do a massive set of correlations of all 2000 variables in the SCCS with the target kinship structures. They state that more sophisticated approaches would be desirable but data insufficiencies preclude this approach. Nonetheless they present a list of so-called explanatory variables that again do not have any controls for historical relatedness or geographical proximity. I have no objection to brute-force exploratory analyses, but this is one area where some theoretically-motivated predictions would have been more desirable than the list in Table 3 with post-hoc considerations.

I hope that the authors can repackage this very interesting work for a more suitable venue.

Decision letter (RSPB-2021-1136.R0)

13-Oct-2021

Dear Mr Itao:

I am writing to inform you that your manuscript RSPB-2021-1136 entitled "Emergence of Kinship Structures and Descent Systems: Multi-level Evolutionary Simulation and Empirical Data Analyses" has, in its current form, been rejected for publication in Proceedings B.

This action has been taken on the advice of referees, who have recommended that substantial revisions are necessary. With this in mind we would be happy to consider a resubmission, provided the comments of the referees are fully addressed. However please note that this is not a provisional acceptance.

The resubmission will be treated as a new manuscript. However, we will approach the same reviewers if they are available and it is deemed appropriate to do so by the Editor. Please note that resubmissions must be submitted within six months of the date of this email. In exceptional circumstances, extensions may be possible if agreed with the Editorial Office. Manuscripts submitted after this date will be automatically rejected.

Please find below the comments made by the referees, not including confidential reports to the Editor, which I hope you will find useful. If you do choose to resubmit your manuscript, please upload the following:

1) A 'response to referees' document including details of how you have responded to the comments, and the adjustments you have made.
2) A clean copy of the manuscript and one with 'tracked changes' indicating your 'response to referees' comments document.
3) Line numbers in your main document.
4) Data - please see our policies on data sharing to ensure that you are complying (https://royalsociety.org/journals/authors/author-guidelines/#data).

To upload a resubmitted manuscript, log into http://mc.manuscriptcentral.com/prsb and enter your Author Centre, where you will find your manuscript title listed under "Manuscripts with Decisions." Under "Actions," click on "Create a Resubmission." Please be sure to indicate in your cover letter that it is a resubmission, and supply the previous reference number.

Sincerely,
Dr Locke Rowe
mailto: proceedingsb@royalsociety.org

Associate Editor
Board Member: 1
Comments to Author:
This manuscript sounds interesting and I think the topic is one that might be suitable for Proc B. That said, I must confess that (like Ref 1) I had a very hard time understanding the details of the paper. I really didn't feel as though I could properly assess its contribution because of this lack of clarity. This is all the more strange given that Ref 2 found the manuscript exceptionally clear.

As just a few examples, on L28-45 I had a very hard time understanding what the C's are (they are never really properly defined). These seem critically important. Likewise, L48-L61 talks of things like double descent etc that, again, are not clearly defined. Likewise, L123+ says this a revised model but I can't tell how it has been revised or why. These are simply a few examples but overall I found the entire manuscript unclear and difficult to follow. As a result, I was left struggling to piece together both the point of the paper and how it came to its conclusions. My view is that the manuscript would need a very substantial revision before it might be suitable for Proc B. If the authors are given a chance to resubmit I think they really need to keep in mind that the readership of Proc B is very broad and so the paper needs to written as such. Otherwise, I think it would be more suitable for a specialized anthropological journal.

Reviewer(s)' Comments to Author:
Referee: 1
Comments to the Author(s)
This paper addresses an interesting and important topic, but it is extremely hard to understand. This is especially a problem in simulation studies because the causal machinery is not so easy to see. Here even the most basic assumptions are hard to figure out, so I recommend the authors be asked to undertake a major revision with the goal of explicating the structure of their model and providing intuitions for the basic results.

Here is a list of some of the things that need to be clarified:

1. Is their migration between groups? Migration is crucial for the maintenance of variation among groups which is in turn crucial for multi-level selection. When traits are discrete, the rare type disadvantage selection assume will act to increase variation among groups. But in the model behavioral and preference traits vary continuously, and this means that even a small amount of migration will homogenize groups. This is crucial, I think, but I do not find any mention of migration in the text.

2. The relationship between the preference structures specified by the inherited traits and the marriage rules is not clear. The results are not so interesting if the marriage rules (not preferences) are imposed exogenously, but I can't figure out exactly what is going on.

3. A crucial result is that evolution creates preference structure so that women in one family prefer the trait (t value) in other families. This seems to be the outcome of simulations, but the paper gives no intuition for why this should be the case.
4. Much data suggests that parents are relatively unimportant in cultural transmission, so the assumption of pure vertical transmission should be tested. This could strengthen the model because if kids learn from other same sex family members, this will create the cultural analog of inbreeding (e.g. you learn from dad and dad's relatives who learned from culturally related members of the previous generation). This will tend to increase between family variation all other things being equal.

Referee: 2
Comments to the Author(s)
I have uploaded my review as a PDF to preserve small bits of notation.

Referee: 3
Comments to the Author(s)
My thanks to the authors and editors for this patience on a review for this paper.

As an evolutionary anthropologist interested in the evolution of human kinship I was pleased to be offered this paper to review. I think there are some excellent ideas here and I am delighted to

see the use of ABMs alongside the empirical ethnographic data. However, I do not think it is suitable for PRSB for a few reasons I will explain briefly below.

The paper explores if various social, cultural and family-level parameters can produce kinship structures akin to the categories proposed by Levi-Strauss's alliance models. While very interesting and important to human kinship specialists, the paper is not motivated to be of interest to a wide evolution/biology/behaviour audience. Kinship is a complex topic in anthropology, and to be of broad interest beyond anthropologists the authors needed to make clear how understanding L-S's structures might shed more general light on human sociality, acting and cooperation. The structures themselves are taken-for-granted, and while they have been influential in anthropology since L-S's work in the 1950s, they are but one of a range of ways to categorise kinship arrangements in human societies. For a PRSB audience, we need a theoretical account of social behaviours grounded in evolutionary principles, and while the parameters in the ABM model could definitely be seen in that way the manuscript does not make the motivation for the model clear.

Despite very much being invested in understanding the work presented, I am sorry to say that I found this paper very hard to comprehend. From the outset it was not clear whether the existence of clans, or the existence of alliance structures, was the target of the model. When I tried to work my way through the specifics of the model I couldn't understand why there was multi-level population structure (families and societies) but no kinship organisation. There is strong evidence from our primate cousins that early hominids would have had some form of kinship structure (see work by e.g. Silk 2021 or Chapais 2008). Comparative cross-cultural work shows us that foraging societies, to the extent that they can provide a picture of the range of pre-Neolithic social structures, have flexibility built into their structures. But I wasn't sure what extent of human history the modelling was meant to provide an explanation for - the original emergence of alliance structures, or their repeated change over cultural evolutionary time? This last point speaks to a wider issue, which is that the manuscript does not have any ethnographic examples to illustrate the phenomena being explained. To speak to an audience beyond modellers, I think this is quite crucial.

I reviewed from the perspective of a cross-cultural evolutionary anthropologist who studies kinship. I am familiar with and can follow ABMs, but I cannot attest to the rigour or quality of the simulation models and analytical approaches presented here -- they might be exemplary but it would be remiss of me to claim I can judge their accuracy. One point of particular difficulty for me was the "cultural traits" that were crucial to the ABM. Families had these traits, and had preferences for them. Some examples were given -- surnames, totems, and occupations -- all of which are phenomena which may denote clan membership in some societies, and this felt circular. I was very unclear on whether these traits could be any kind of shared public signifier, and whether they could only be symbolic. Occupation, for example, signals information about resources as well as social desirability.
I do think there is too much packed into this paper. It is the kind of work that would be better in another venue as a two-part paper Part 1: Model - Part 2: Empirical analyses, with ample space given to motivate the investigations and to describe and justify the parameters under study. The authors have made a valiant attempt at compressing a lot of work into a PRSB format but it is at the expense of clarity.

Finally, I felt on more familiar terrain with the final cross-cultural analyses in the paper, and I offer some specific remarks here which I hope the authors can use to improve their study.

* The section "Classification of Kinship Structures" in the Methods could be much clearer. Interpreting the SM tables and values of variables requires deep specialist knowledge of kinship jargon and worked ethnographic examples would be more tractable.

* Only 60/186 societies were included, because of the exclusive definitions of the four structures. The 186 societies in the SCCS are, to a first approximation, the most ethnographically well-

described communities that anthropologists have studies, but L-S's framework can only account for kinship practices in a third of them. This weakens any claim of "universal anthropology" (the authors' choice of keywords).

* Because only 60 were identified, I was less convinced by the "matching" data presented in Figure 4. This is not only because of the small sample size, but because of phylogenetic and spatial autocorrelation effects. These still exist in the SCCS, despite wishful-thinking claims to the contrary.

* The authors then looked for explanatory variables for the kinship structures (line 340): "we conducted Spearman’s rank correlations 340 between the SCCS variables and the structures." I think what they have done here is to do a massive set of correlations of all 2000 variables in the SCCS with the target kinship structures. They state that more sophisticated approaches would be desirable but data insufficiencies preclude this approach. Nonetheless they present a list of so-called explanatory variables that again do not have any controls for historical relatedness or geographical proximity. I have no objection to brute-force exploratory analyses, but this is one area where some theoretically-motivated predictions would have been more desirable than the list in Table 3 with post-hoc considerations.

I hope that the authors can repackage this very interesting work for a more suitable venue.

Author's Response to Decision Letter for (RSPB-2021-1136.R0)

See Appendix B.

RSPB-2021-2641.R0

Review form: Reviewer 2

Recommendation
Accept with minor revision (please list in comments)

Scientific importance: Is the manuscript an original and important contribution to its field?
Good

General interest: Is the paper of sufficient general interest?
Good

Quality of the paper: Is the overall quality of the paper suitable?
Good

Is the length of the paper justified?
Yes

Should the paper be seen by a specialist statistical reviewer?
No

Do you have any concerns about statistical analyses in this paper? If so, please specify them explicitly in your report.
No

It is a condition of publication that authors make their supporting data, code and materials available - either as supplementary material or hosted in an external repository. Please rate, if applicable, the supporting data on the following criteria.

Is it accessible?
Yes

Is it clear?
N/A

Is it adequate?
N/A

Do you have any ethical concerns with this paper?
No

Comments to the Author
I have just read the revision of manuscript RSPB-2021-1136, now numbered RSPB-2021-2641. I believed this was an interesting and valuable contribution in the initial round of review, and I still believe that now. Moreover, I believe the manuscript itself has improved in revision. I have only minor suggestions for further improvement. Of course, the authors may take or leave these as they see fit.

Line 2: "form the basic units of society…": Should "form" be "shape?" To me, "form" implies that marriage and descent somehow constitute the basic units of society (ie, families), but I think the authors means that they determine how families arise and behave.

Lines 8-9: "Marriage and descent relationships are, thus, determined by clan attributions": Maybe "Marriage and descent relationships, and also kinship, are determined by clan attributions?" For me as a biologist, kinship implies genetic relatedness, but I think that is not necessarily true here, and it took me a while to understand that members of the same clan who are not genetically related are still "kin" in this study. Or, if I am wrong about this, then maybe a very clear definition of "kinship" would help!

Line 55: Should be "…and the paternally…"

Line 69: Should "dominant over" be "more common than?" For me, "dominant over" implies that when two things interact, the dominant one will somehow prevail. But, it is not clear to me that there is any interaction or prevailing in the empirical systems. (Perhaps there is in the model, but that is not what the authors are describing here.)

Line 72-73: "distinction of cultural associations": I do not understand what this means.

Line 92: Is "indigenous" the right word here? Maybe "self-organising?"

Line 124: I think "unveil" is too strong a word here. Maybe "suggest?"

Line 157: "by eliminating it": Maybe "by eliminating lineages?" It was not 100% clear to me what "it" referred to in this sentence.

Line 189: Should "supposed" be "assumed?" That is, this is a specific assumption of this model, and not a consensus opinion about empirical systems based on previous research, right?

Figure 1B: This figure confused me a little. Would there not also be competition between two families with the same preference AND the same trait value? If so, I would expect an arrow from

the top left family that loops back to itself. But, if each circle represents just one family rather than a class of families, then maybe this would be even more confusing, because the authors do not want to imply that families compete with themselves. Also, I was a little confused by the grid-like layout of the families in 1B. This seems to imply that traits and preferences are discrete, but in practice (at least at the outset of the model, which is what the authors are describing here) they are continuous. All in all, I am not sure whether figure 1B makes the model clearer or less clear. One thing that might help is if the authors could be very clear at this point in the text (maybe the paragraph beginning on line 206) that every family is both a friend (based the similarity of their traits, or the match between their traits and preferences) and a rival (based on the similarity of their preferences) to every other. This becomes clear later in the manuscript, but would have helped me here.

Line 240: "without introducing any rules": I might say "starting from an initially homogenous state." After all, there are MANY rules (eg, about how families compete, how and when marriages form, etc).

Line 255-256: "Siblings belonged to the same cluster." Maybe specify "at birth," since this need not be true after marriage? (This probably seems pedantic, but it really did confuse me for a minute, and I wondered if maybe the authors were referring to sibling families.)

Line 285: Perhaps specify that a matrilineal descent system evolves in this particular simulation, but a patrilineal system might evolve in others? (I think this becomes clearer later, but might help readers here.)

Lines 364-367: I was confused here by whether the authors were referring to the model or to real systems. I think the word "realistically" is meant to make that clear, but it did not fully work for me. Maybe "In real systems…"

Lines 380-382: "Forty societies were … marriage only." Why are these societies not examples of generalised exchange? From the introduction I thought they should be.

Line 570 "mating competition or niche construction." I might say "resource competition and evolved mate preferences." The authors might want to have a look at Bolnick and Doebli 2003 (Evolution), which includes an interesting case where disassortative mating (ie, a preference for mates with traits dissimilar to one's own) leads to speciation (see figure 8). This seems similar to the restricted exchange with double descent that the authors obtain here. I think most biologists agree that this is an implausible outcome in speciation models, but as the authors explain, human cultural evolution does not follow exactly the same rules. Personally I find it interesting that an idiosyncratic mechanism in Bolnick and Doebeli's work might reflect an important mechanism in this new context.

Lines 580-583: "As population size … is prohibited." Again, this is in real systems, right? Perhaps specify just to avoid any confusion.

Line 611-612: "we have demonstrated that various kinship structure emerge": In the model, correct? If so, perhaps specify. As the authors explain above, more work needs to be done to determine whether these same mechanisms have really shaped human kinship structures. To me, much of the value of this paper is that it invites this further work!

Tucker Gilman
3 January 2022

Decision letter (RSPB-2021-2641.R0)

18-Jan-2022

Dear Mr Itao

I am pleased to inform you that your manuscript RSPB-2021-2641 entitled "Emergence of Kinship Structures and Descent Systems: Multi-level Evolutionary Simulation and Empirical Data Analysis" has been accepted for publication in Proceedings B.

The referee(s) have recommended publication, but also suggest some minor revisions to your manuscript. Therefore, I invite you to respond to the referee(s)' comments and revise your manuscript. Because the schedule for publication is very tight, it is a condition of publication that you submit the revised version of your manuscript within 7 days. If you do not think you will be able to meet this date please let us know.

To revise your manuscript, log into https://mc.manuscriptcentral.com/prsb and enter your Author Centre, where you will find your manuscript title listed under "Manuscripts with Decisions." Under "Actions," click on "Create a Revision." Your manuscript number has been appended to denote a revision. You will be unable to make your revisions on the originally submitted version of the manuscript. Instead, revise your manuscript and upload a new version through your Author Centre.

When submitting your revised manuscript, you will be able to respond to the comments made by the referee(s) and upload a file "Response to Referees". You can use this to document any changes you make to the original manuscript. We require a copy of the manuscript with revisions made since the previous version marked as ‘tracked changes’ to be included in the ‘response to referees’ document.

Before uploading your revised files please make sure that you have:

1) A text file of the manuscript (doc, txt, rtf or tex), including the references, tables (including captions) and figure captions. Please remove any tracked changes from the text before submission. PDF files are not an accepted format for the "Main Document".

2) A separate electronic file of each figure (tiff, EPS or print-quality PDF preferred). The format should be produced directly from original creation package, or original software format. PowerPoint files are not accepted.

3) Electronic supplementary material: this should be contained in a separate file and where possible, all ESM should be combined into a single file. All supplementary materials accompanying an accepted article will be treated as in their final form. They will be published alongside the paper on the journal website and posted on the online figshare repository. Files on figshare will be made available approximately one week before the accompanying article so that the supplementary material can be attributed a unique DOI.

Online supplementary material will also carry the title and description provided during submission, so please ensure these are accurate and informative. Note that the Royal Society will not edit or typeset supplementary material and it will be hosted as provided. Please ensure that the supplementary material includes the paper details (authors, title, journal name, article DOI). Your article DOI will be 10.1098/rspb.[paper ID in form xxxx.xxxx e.g. 10.1098/rspb.2016.0049].

4) A media summary: a short non-technical summary (up to 100 words) of the key findings/importance of your manuscript.

5) Data accessibility section and data citation
It is a condition of publication that data supporting your paper are made available either in the electronic supplementary material or through an appropriate repository (https://royalsociety.org/journals/authors/author-guidelines/#data).

In order to ensure effective and robust dissemination and appropriate credit to authors the dataset(s) used should be fully cited. To ensure archived data are available to readers, authors should include a 'data accessibility' section immediately after the acknowledgements section. This should list the database and accession number for all data from the article that has been made publicly available, for instance:
• DNA sequences: Genbank accessions F234391-F234402
• Phylogenetic data: TreeBASE accession number S9123
• Final DNA sequence assembly uploaded as online supplemental material
• Climate data and MaxEnt input files: Dryad doi:10.5521/dryad.12311
NB. From April 1 2013, peer reviewed articles based on research funded wholly or partly by RCUK must include, if applicable, a statement on how the underlying research materials – such as data, samples or models – can be accessed. This statement should be included in the data accessibility section.

If you wish to submit your data to Dryad (http://datadryad.org/) and have not already done so you can submit your data via this link http://datadryad.org/submit?journalID=RSPB&manu=(Document not available) which will take you to your unique entry in the Dryad repository. If you have already submitted your data to dryad you can make any necessary revisions to your dataset by following the above link. Please see https://royalsociety.org/journals/ethics-policies/data-sharing-mining/ for more details.

6) For more information on our Licence to Publish, Open Access, Cover images and Media summaries, please visit https://royalsociety.org/journals/authors/author-guidelines/.

Once again, thank you for submitting your manuscript to Proceedings B and I look forward to receiving your revision. If you have any questions at all, please do not hesitate to get in touch.

Sincerely,
Dr Locke Rowe
mailto: proceedingsb@royalsociety.org

Associate Editor
Board Member
Comments to Author:
none

Reviewer(s)' Comments to Author:
Referee: 2
Comments to the Author(s).
I have just read the revision of manuscript RSPB-2021-1136, now numbered RSPB-2021-2641. I believed this was an interesting and valuable contribution in the initial round of review, and I still believe that now. Moreover, I believe the manuscript itself has improved in revision. I have only minor suggestions for further improvement. Of course, the authors may take or leave these as they see fit.

Line 2: "form the basic units of society…": Should "form" be "shape?" To me, "form" implies that marriage and descent somehow constitute the basic units of society (ie, families), but I think the authors means that they determine how families arise and behave.

Lines 8-9: “Marriage and descent relationships are, thus, determined by clan attributions”: Maybe “Marriage and descent relationships, and also kinship, are determined by clan attributions?” For me as a biologist, kinship implies genetic relatedness, but I think that is not necessarily true here, and it took me a while to understand that members of the same clan who are not genetically related are still “kin” in this study. Or, if I am wrong about this, then maybe a very clear definition of “kinship” would help!

Line 55: Should be “…and the paternally…”

Line 69: Should “dominant over” be “more common than?” For me, “dominant over” implies that when two things interact, the dominant one will somehow prevail. But, it is not clear to me that there is any interaction or prevailing in the empirical systems. (Perhaps there is in the model, but that is not what the authors are describing here.)

Line 72-73: “distinction of cultural associations”: I do not understand what this means.

Line 92: Is “indigenous” the right word here? Maybe “self-organising?”

Line 124: I think “unveil” is too strong a word here. Maybe "suggest?"

Line 157: “by eliminating it”: Maybe “by eliminating lineages?” It was not 100% clear to me what “it” referred to in this sentence.

Line 189: Should “supposed” be “assumed?” That is, this is a specific assumption of this model, and not a consensus opinion about empirical systems based on previous research, right?

Figure 1B: This figure confused me a little. Would there not also be competition between two families with the same preference AND the same trait value? If so, I would expect an arrow from the top left family that loops back to itself. But, if each circle represents just one family rather than a class of families, then maybe this would be even more confusing, because the authors do not want to imply that families compete with themselves. Also, I was a little confused by the grid-like layout of the families in 1B. This seems to imply that traits and preferences are discrete, but in practice (at least at the outset of the model, which is what the authors are describing here) they are continuous. All in all, I am not sure whether figure 1B makes the model clearer or less clear. One thing that might help is if the authors could be very clear at this point in the text (maybe the paragraph beginning on line 206) that every family is both a friend (based the similarity of their traits, or the match between their traits and preferences) and a rival (based on the similarity of their preferences) to every other. This becomes clear later in the manuscript, but would have helped me here.

Line 240: “without introducing any rules”: I might say “starting from an initially homogenous state.” After all, there are MANY rules (eg, about how families compete, how and when marriages form, etc).

Line 255-256: “Siblings belonged to the same cluster.” Maybe specify “at birth,” since this need not be true after marriage? (This probably seems pedantic, but it really did confuse me for a minute, and I wondered if maybe the authors were referring to sibling families.)

Line 285: Perhaps specify that a matrilineal descent system evolves in this particular simulation, but a patrilineal system might evolve in others? (I think this becomes clearer later, but might help readers here.)

Lines 364-367: I was confused here by whether the authors were referring to the model or to real systems. I think the word “realistically” is meant to make that clear, but it did not fully work for me. Maybe “In real systems…”

Lines 380-382: “Forty societies were … marriage only.” Why are these societies not examples of generalised exchange? From the introduction I thought they should be.

Line 570 “mating competition or niche construction.” I might say "resource competition and evolved mate preferences." The authors might want to have a look at Bolnick and Doebli 2003 (Evolution), which includes an interesting case where disassortative mating (ie, a preference for mates with traits dissimilar to one’s own) leads to speciation (see figure 8). This seems similar to the restricted exchange with double descent that the authors obtain here. I think most biologists agree that this is an implausible outcome in speciation models, but as the authors explain, human cultural evolution does not follow exactly the same rules. Personally I find it interesting that an idiosyncratic mechanism in Bolnick and Doebeli’s work might reflect an important mechanism in this new context.

Lines 580-583: “As population size … is prohibited.” Again, this is in real systems, right? Perhaps specify just to avoid any confusion.

Line 611-612: “we have demonstrated that various kinship structure emerge”: In the model, correct? If so, perhaps specify. As the authors explain above, more work needs to be done to determine whether these same mechanisms have really shaped human kinship structures. To me, much of the value of this paper is that it invites this further work!

Tucker Gilman
3 January 2022

Author's Response to Decision Letter for (RSPB-2021-2641.R0)

See Appendix C.

Decision letter (RSPB-2021-2641.R1)

25-Jan-2022

Dear Mr Itao

I am pleased to inform you that your manuscript entitled "Emergence of Kinship Structures and Descent Systems: Multi-level Evolutionary Simulation and Empirical Data Analysis" has been accepted for publication in Proceedings B.

You can expect to receive a proof of your article from our Production office in due course, please check your spam filter if you do not receive it. PLEASE NOTE: you will be given the exact page length of your paper which may be different from the estimation from Editorial and you may be asked to reduce your paper if it goes over the 10 page limit.

If you are likely to be away from e-mail contact please let us know. Due to rapid publication and an extremely tight schedule, if comments are not received, we may publish the paper as it stands.

If you have any queries regarding the production of your final article or the publication date please contact procb_proofs@royalsociety.org

Your article has been estimated as being 10 pages long. Our Production Office will be able to confirm the exact length at proof stage.

Data Accessibility section
Please remember to make any data sets live prior to publication, and update any links as needed when you receive a proof to check. It is good practice to also add data sets to your reference list.

Open Access
You are invited to opt for Open Access, making your freely available to all as soon as it is ready for publication under a CCBY licence. Our article processing charge for Open Access is £1700. Corresponding authors from member institutions (http://royalsocietypublishing.org/site/librarians/allmembers.xhtml) receive a 25% discount to these charges. For more information please visit http://royalsocietypublishing.org/open-access.

Paper charges
An e-mail request for payment of any related charges will be sent out shortly. The preferred payment method is by credit card; however, other payment options are available.

Electronic supplementary material:
All supplementary materials accompanying an accepted article will be treated as in their final form. They will be published alongside the paper on the journal website and posted on the online figshare repository. Files on figshare will be made available approximately one week before the accompanying article so that the supplementary material can be attributed a unique DOI.

You are allowed to post any version of your manuscript on a personal website, repository or preprint server. However, the work remains under media embargo and you should not discuss it with the press until the date of publication. Please visit https://royalsociety.org/journals/ethics-policies/media-embargo for more information.

Thank you for your fine contribution. On behalf of the Editors of the Proceedings B, we look forward to your continued contributions to the Journal.

Sincerely,
Proceedings B
mailto: proceedingsb@royalsociety.org

Appendix A

I have reviewed manuscript RSPB-2021-1136, *Emergence of Kinship Structures and Descent Systems: Multi-level Evolutionary Simulation and Empirical Data Analyses*. In this manuscript, the authors propose a model in which trait values and preferences for trait values in a mate selection context are socially transmitted within families in a population. Trait values and preferences in families and in the population can evolve, and the values that evolve can lead to the emergence of different kinship structures and descent systems. The authors show that which kinship structures and descent systems evolve depends on the relative strengths of cooperation (among kin) and competition (among rivals for mates) in the population. They analyse the model numerically and analytically, and they argue that their results match the empirical data.

I should admit here that I am a biological theoretician, not an anthropologist, so while I am familiar with modelling approaches, the precise systems being studied are outside my area of expertise. Nonetheless, I found the manuscript to be clear and well written. I had to go back and forth from the body of the paper to the methods to fully understand the model, and there are a few places where I think the authors could make the model clearer for their readers. However, I found the results to be extremely well presented. The introduction and discussion provided the information I needed to understand the work without unnecessary digressions. In all, I found the manuscript interesting and enjoyable to read. I believe this work can help organize and formalize the way researchers think about the big questions the authors have addressed, and I think it would be a good fit for Proceedings B.

For me, this manuscript opened more questions than it answered. It appears to me that the mechanisms in the paper are in some ways analogous to those in the ecological speciation literature. That is, when non-random mating is possible, competition can lead to population divergence, and stronger competition can lead to the emergence of a larger number of phenotypically diverged groups. Michael Doebeli and collaborators have produced excellent work in this area. The analogy to speciation made me wonder whether the predictions would be different if the strengths of cooperation and competition (controlled by τ in the model) could evolve independently (perhaps by linking to the ideas of altruism and spite). I also wondered whether the results are sensitive to the number of independent traits in the model. In my opinion, the existence of open questions is an additional argument for publishing this work.

In my comments below, I have made several suggestions for places where I think the authors could make the manuscript easier to understand and more accessible to readers. As always, the authors can take or leave my advice as they see fit. Most of the suggestions are minor, and I would not expect lengthy responses to any of them. Finally, while I have suggested additional discussion in places, I encourage the authors to keep that as brief as practical. One of strengths of this manuscript is that it is concise!

General comments:

1. The authors argue that the mate selection cycle that evolves in figure 2C represents generalized exchange. I agree that the mate selection cycle is described by the same C_m>2 criterion, but the structure that evolves seems to be quite special. I would think that in empirically realized generalized exchange, individuals might choose mates

from any clan (or at least from any one of a number of clans) other than their own. Perhaps the easiest way for this to arise would be a simple self-avoidance mechanism, where the phenotype p tells individuals which mates to avoid (e.g., members of their own family) rather than which to choose. In this case, it is not intuitive to me that this would lead to the emergence of exactly three (or indeed any) distinct clans. Avoidance (or negative preference) cannot arise in this model, and I am not suggesting the authors should change the model to allow it. But, it might be worth briefly discussing whether the form of generalized exchange that arises in this system really corresponds to that in empirical systems.

2. Similar to my concerns about generalized exchange, I am not convinced that the system that the authors call an "incest structure" really reflects empirically realized incest structures. In the authors' incest structures, there are no clans at all, so it is hard to argue that mating occurs within clans. In figure 2A, the family traits and preferences do not line up on the $t_1=p_1$ and $t_2=p_2$ axes, so there does not seem to be a preference for incest within families. In fact, there might even be avoidance of incest within families – we cannot really tell without seeing the relationship between $t_1 - p_1$ and $t_2 - p_2$. Moreover, we might wonder if the incest structures that emerge in this model achieve the same trait topology as we might expect from empirical incest structures. For example, I can imagine a case where a preference for incest (i.e., mating within clans, not necessarily within families) allows clan traits to diverge in preference space, so reducing competition. In such cases, some forms of strong competition for mates might even favor the evolution of within-clan mating (e.g., if clans have a mechanism for suppressing internal competition, but not external competition). On the other hand, in the authors' model, where there is no mechanism for suppressing within-clan competition, clans with high incest might be destabilized by internal competition. Perhaps this could partly explain why empirical incest systems do not match the model predictions as well as the other kinship structures? (But, all of my thoughts here may result from my own poor understanding of empirical incest structures!)

3. One interesting characteristic of this model is that (if I understand correctly), the traits and preferences we are studying are either strictly paternally or strictly maternally inherited, and there is no mixing or blending between them. So, an individual acquires traits (with error) from its paternal grandfather and its maternal grandmother, but there is no influence of its paternal grandmother or maternal grandfather (except of course that they influence whether the individual exists at all). There cases where this may be approximately true (e.g., surnames in some cultures), but others where some degree of blending may occur. The authors mention this in the discussion, albeit indirectly. However, it might help readers if the assumption of strict maternal and paternal trait acquisition were presented clearly very early in the manuscript. I think this would have helped me to understand the model more easily.

4. I was, and remain to some degree, confused by time in this model. If a man and woman marry, then they form a new family, and that family (may) produce offspring in the next time step. The offspring have two time steps to marry, and if they remain

unmarried then they are removed from the population. So, in this sense, time steps seem to correspond to generations. It is perhaps slightly odd that individuals can start families a full generation after they reach marriageable age, but this may be plausible for cultures with short generation times. But, is it true that the married pair is not removed from the population after they have reproduced, or in any subsequent time step? So, in effect, once married an couple can potentially persist and reproduce forever? If so, this seems very strange, and it might or might not matter for the model. A married pair that is 50 generations old might still be passing on traits and preferences from a much earlier form of its clan, and this might impede evolution, or might help a clan or society persist when it would otherwise disappear. Even if I am correct about this, I am not sure I would rerun the model to fix it (unless the authors had to rerun it for something else – then maybe it would be worth thinking about). But, it would help readers if the authors could explain time and the persistence of families in the model very clearly.

Line-by-line comments:

Line 7: This use of “attribution” was new to me, and I initially thought it meant “attributes.” It might help non-specialist readers if the first use of attribution said something like “clan attribution (i.e., the clan to which they belong).”

Lines 28-47: The formalism of describing kinship structures by (C_m,C_d) was also new to me, but is very important in this manuscript. I struggled to understand some of these ideas, particularly $C_d = 2$. I initially thought this meant that the children of mother A and father B would join some new clan to which neither parent belonged, but then that the grandchildren would somehow revert back to the clan of their grandfather B. But, I now think that the children somehow associate with different clans (sometimes the mother’s and sometimes the father’s) for different purposes, and that $C_d = 2$ indicates that the child half belongs to the father’s clan (so the mean cumulative return time of a male descendent to the male progenitor’s clan is 2). In any case, I think this could be more clearly explained. This is important because my failure to fully understand this idea initially later caused me to struggle with the idea of double descent in the model results.

Lines 48-50: I did not understand this sentence, possibly because I did not understand what “are referred to” meant.

Lines 113-116: The introduction focuses on clans. When I reached these lines, I was confused about whether families or societies correspond to clans. It might help to tell the reader right here (or otherwise very early on) that clans are not initially created, but are instead an emergent property of the system.

Line 129: I struggled with $|t_i-p_j|/\tau$, for an admittedly naïve reason. For mathematicians, the absolute value of a difference in vectors is a scalar, but in most computational languages it is still a vector. So, while the authors’ notation is absolutely correct, I initially read this as being a vector. This caused me even more problems later when I was trying to understand how traits are transmitted. It might be worth reminding readers with poor mathematical

training (like me!) that $|t_i - p_j|$ is just the Euclidean distance between the male trait combination and the female preference combination.

Lines 150-151: "At larger d_c values, a small proportion of non-cooperators is crucial for survival." I initially read this to mean that, for some reason, families need at least a small number of non-cooperators to survive, but I think it means instead that they must have no more than a small number of non-cooperators if they are to survive. This could be stated more clearly.

Figures 2 and 3: I am not sure I fully understand what is being represented here. In the legend to figure 3, the authors say they conducted "over 100 trials for each condition." But, what are the "conditions?" Presumably they are combinations of d_c, d_m, N_s, N_f, and μ. If so, what set of conditions was studied? I could probably guess by counting the squares in the figures (8 x 8?), but I would rather be told. And, how many is "over 100?" And, since each of these trials includes multiple societies, does that mean the real number of societies studied (since these are the levels at which kinship structure emerge) is >5000? (I imagine we can assume that the >50 societies generated by a single trial are effectively independent. I might be worth a quick check just to be sure.) Finally, in figure 2, are the societies shown just illustrative examples from the full set of societies and trials? That is, were they chosen because they look particularly good rather than in any sort of systematic or random way? (That is fine of course, but I would like to know!)

Lines 325-389: I struggled with the empirical analysis more than with the model. Am I correct in believing that the authors looked at the set of SCCS variables and determined by knowledge of the system or by intuition (and hopefully *before* studying any correlations!!) whether each one was related to d_c or d_m? Then, after this, they asked whether each of these variables was correlated in the expected way (according to the authors' assessment of its role in d_c or d_m) with the empirical kinship structures, and this value is reported in table 3? Then, finally, they computed $\widetilde{d_c}$ and $\widetilde{d_m}$ values for each empirical population by averaging over the standardized and equally weighted variables that they believe contribute to d_c and d_m, and they plotted the empirical kinship structures as a function of $\widetilde{d_c}$ and $\widetilde{d_m}$ in figure 3B? If so, I am fine with all of this. But, I think the authors might make this easier to understand by explaining the assignment of SCCS traits to d_c or d_m first, and (if it is true) emphasizing that this was done before studying the correlations between traits and kinship structures. If the assignment of traits to d_c or d_m was not *a priori*, I still do not think it is fatal to the authors' argument, but it would influence how much confidence I would have in the assignments and in the empirical results for future work, so it is important to report.

Line 690: "Peter, T" should be "Turchin, P."

Tucker Gilman
18 August 2021

Appendix B

Point-by-point responses: “Emergence of Kinship Structures and Descent Systems: Multi-level Evolutionary Simulation and Empirical Data Analysis” (RSPB-2021-1136)

Associate Editor
Board Member: 1
Comments to Author: *This manuscript sounds interesting and I think the topic is one that might be suitable for Proc B. That said, I must confess that (like Ref 1) I had a very hard time understanding the details of the paper. I really didn’t feel as though I could properly assess its contribution because of this lack of clarity. This is all the more strange given that Ref 2 found the manuscript exceptionally clear.*

>>
We thank you for your positive evaluation and suggested revisions. Following your and the reviewers’ suggestions, we have accordingly updated the paper to provide a better explanation.

As just a few examples, on L28-45 I had a very hard time understanding what the C’s are (they are never really properly defined). These seem critically important. Likewise, L48-L61 talks of things like double descent etc that, again, are not clearly defined. Likewise, L123+ says this a revised model but I can’t tell how it has been revised or why. These are simply a few examples but overall I found the entire manuscript unclear and difficult to follow. As a result, I was left struggling to piece together both the point of the paper and how it came to its conclusions. My view is that the manuscript would need a very substantial revision before it might be suitable for Proc B. If the authors are given a chance to resubmit I think they really need to keep in mind that the readership of Proc B is very broad and so the paper needs to written as such. Otherwise, I think it would be more suitable for a specialized anthropological journal.

>>
Thank you for articulating such important suggestions. Accordingly, we revised the explanation of marriage and descent cycles in lines 21–33. Marriage and descent cycles are defined as the length of cycles composed by these two relationships. For example, if a rule exists for women in clan X to marry men in clan Y, the marriage relationship is represented by $X \Rightarrow Y$. If a limited number of clans exist in the society, the whole relationships of clans should be $X \Rightarrow Y \Rightarrow \cdots \Rightarrow X$. Here, marriage relationships of clans constitute a cycle, the length of which is termed the marriage cycle C_m (e.g., $C_m = 3$ if $X \Rightarrow Y \Rightarrow Z \Rightarrow X$). Similarly, if the children belong to clan B, and their father to clan A, it represents the descent relationship $A \rightarrow B$. This relationship also constitutes the cycle, and its length is termed the descent cycle C_d.

We also explained descent systems in lines 51–66. When children belong to the same clan as either their father or mother, the descent system is classified as patrilineal or matrilineal, respectively. In these cases, $C_d = 1$, and paternally or maternally inherited trait is significant for characterising clans. Conversely, when $C_d > 1$, children inherit cultural traits from both parents independently and have attributions different from either parent. When both paternally and maternally inherited traits are significant for characterising clans, the system is termed double descent. In the above cases, traits are assumed to be independently inherited through paternal and maternal lines.

The model has been revised from the previous one in the following points: We eliminated the intermediate layer of “lineages” between families and societies to increase clarity and make the model suitable for analytical calculation. Further, we had previously assumed that the first dimensional values of traits and preferences t_1 and p_1 are inherited from the father, and the second dimensional values t_2 and p_2 are inherited either from the father or the mother, depending on families’ strategies. This assumption had biased the model towards paternal inheritance, and limited the evolution of descent systems, as the matrilineal descent system is set to be harder to evolve. Hence, we revised this to enable us to discuss the evolution of various descent systems. With these revisions, the simplicity and the generality of the model is expanded to fit the broad readership of Proceedings B. We have stated these in lines 154–158 and 182–191.

We consider the cooperation among kin and mates, and the competition among mating rivals. By introducing the evolutionary pressure to increase cooperation among kin and mates, and to decrease competition among rivals, we illustrated that diverse kinship structures evolve depending on the pressures. With the pressure to increase cooperators by increasing kin and mates, isolated families without sufficient

friend values are removed, and families are clustered. With the pressure to decrease mating rivals, families' preferences diverge. Accordingly, under sufficient strengths of both pressures, that is, sufficient d_c and d_m values, families form several discrete clusters united by marital relationships in $(\boldsymbol{t}, \boldsymbol{p})$ space. We added this in lines 249–255 and 469–472 and believe that the enhanced explanation will help readers understand our results.

Furthermore, we updated the data analysis and covered more societies (from 60/186 to 146/186 societies in the SCCS). Reviewers 2 and 3 had commented on the data analysis and now, we believe we can overcome the weakness.

Notably, structure formation by cooperation and competition will also gather interest in general readerships in biology, because it is comparable to the mating preference model for speciation, as pointed out by Reviewer 2. We added this explanation in lines 567–575.

Reviewer # 1

0) *This paper addresses an interesting and important topic, but it is extremely hard to understand. This is especially a problem in simulation studies because the causal machinery is not so easy to see. Here even the most basic assumptions are hard to figure out, so I recommend the authors be asked to undertake a major revision with the goal of explicating the structure of their model and providing intuitions for the basic results.*

>>

Thank you for this evaluation and your helpful suggestions for the revision. Accordingly, we have expanded the explanations throughout the manuscript to make them more understandable.

1) *Is their migration between groups? Migration is crucial for the maintenance of variation among groups which is in turn crucial for multi-level selection. When traits are discrete, the rare type disadvantage selection assume will act to increase variation among groups. But in the model behavioural and preference traits vary continuously, and this means that even a small amount of migration will homogenize groups. This is crucial, I think, but I do not find any mention of migration in the text.*

>>

As stated in lines 140—143, we do not consider spatial migration in the main model. Consequently, the variation among societies increases by mutation within each society. However, as we showed in Fig. S1, our results are robust to the inclusion of migration, if it is not too strong.

2) *The relationship between the preference structures specified by the inherited traits and the marriage rules is not clear. The results are not so interesting if the marriage rules (not preferences) are imposed exogenously, but I can't figure out exactly what is going on.*

>>

Thank you for this comment. In our model, the probability of marriage of men in family i and women in family j is proportional to $\exp(-|\boldsymbol{t}^i - \boldsymbol{p}^j|^2/\tau^2)$. In other words, they get married if men's traits are close to women's preferences. Since all families have the same traits and preferences at the initial condition, the rules prohibiting within-clan (group of culturally similar families) marriages are thoroughly emergent properties in our model. Even though the preference rule is provided, the marriage rules that determine the mate clan (other than their own) emerge, depending on the distribution of traits and preferences. We have explained the marriage process in lines 230–234.

3) *A crucial result is that evolution creates preference structure so that women in one family prefer the trait (t value) in other families. This seems to be the outcome of simulations, but the paper gives no intuition for why this should be the case.*

>>

Thank you for this comment. We consider the cooperation among kin and mates, and the competition among mating rivals. By introducing the evolutionary pressure to increase cooperation among kin and mates, and to decrease competition among rivals, we demonstrated that diverse kinship structures evolve depending on the pressures. With the pressure to increase cooperators by increasing kin and mates, isolated families without sufficient friend values were removed, and families become clustered. With the

pressure to decrease mating rivals, families' preferences diverge. Accordingly, under sufficient strengths of both pressures, that is, sufficient d_c and d_m values, families form several discrete clusters united by marital relationships in $(\boldsymbol{t}, \boldsymbol{p})$ space, such that the cooperation is enhanced and the competition is reduced.

We added an intuitive explanation of the dynamics in lines 249–255 in the revised manuscript.

4) *Much data suggests that parents are relatively unimportant in cultural transmission, so the assumption of pure vertical transmission should be tested. This could strengthen the model because if kids learn from other same sex family members, this will create the cultural analog of inbreeding (e.g. you learn from dad and dad's relatives who learned from culturally related members of the previous generation). This will tend to increase between family variation all other things being equal.*

>>
Thank you for your suggestion. First, the cultural traits of families and kinship are known to be slow to change, because they tend to be inherited from parents or close relatives, and are regulated by social norms [1, 2], as clarified in lines 77–83. Then, even if kids learn traits from their parents' close relatives, it will make a minimal difference in the results qualitatively, because the traits are similar to that of their parents. However, as you correctly suggested, the way that traits and preferences are inherited, and how strongly social norms regulate precise inheritance would quantitatively affect the values of μ in our model. Though considering this would be important in estimating μ from real data, we could, however, not accomplish this. We have stated this as a limitation in lines 562–564.

Reviewer # 2
0) *I have reviewed manuscript RSPB-2021-1136, Emergence of Kinship Structures and Descent Systems: Multi-level Evolutionary Simulation and Empirical Data Analyses. In this manuscript, the authors propose a model in which trait values and preferences for trait values in a mate selection context are socially transmitted within families in a population. Trait values and preferences in families and in the population can evolve, and the values that evolve can lead to the emergence of different kinship structures and descent systems. The authors show that which kinship structures and descent systems evolve depends on the relative strengths of cooperation (among kin) and competition (among rivals for mates) in the population. They analyse the model numerically and analytically, and they argue that their results match the empirical data.*
I should admit here that I am a biological theoretician, not an anthropologist, so while I am familiar with modelling approaches, the precise systems being studied are outside my area of expertise. Nonetheless, I found the manuscript to be clear and well written. I had to go back and forth from the body of the paper to the methods to fully understand the model, and there are a few places where I think the authors could make the model clearer for their readers. However, I found the results to be extremely well presented. The introduction and discussion provided the information I needed to understand the work without unnecessary digressions. In all, I found the manuscript interesting and enjoyable to read. I believe this work can help organize and formalize the way researchers think about the big questions the authors have addressed, and I think it would be a good fit for Proceedings B.
For me, this manuscript opened more questions than it answered. It appears to me that the mechanisms in the paper are in some ways analogous to those in the ecological speciation literature. That is, when non-random mating is possible, competition can lead to population divergence, and stronger competition can lead to the emergence of a larger number of phenotypically diverged groups. Michael Doebeli and collaborators have produced excellent work in this area. The analogy to speciation made me wonder whether the predictions would be different if the strengths of cooperation and competition (controlled by t in the model) could evolve independently (perhaps by linking to the ideas of altruism and spite). I also wondered whether the results are sensitive to the number of independent traits in the model. In my opinion, the existence of open questions is an additional argument for publishing this work.
In my comments below, I have made several suggestions for places where I think the authors could make the manuscript easier to understand and more accessible to readers. As always, the authors can take or leave my advice as they see fit. Most of the suggestions are minor, and I would not expect lengthy responses to any of them. Finally, while I have suggested additional discussion in places, I encourage the authors to keep that as brief as practical. One of strengths of this manuscript is that it is concise!

>>
Thank you for your positive evaluation and suggestions for revisions. We have added detailed explanations

in the model section in lines 195–202, 208–210, and 230–234. Regarding the relationship with ecological speciation, our model and its dynamics indeed share some similarities with the mating preference model for sympatric speciation [3]. However, the uniqueness of human kinship lies in the fact that different groups can be united through marriage owing to the development of the ability for kin recognition. Our model, that includes cooperation among mates, exhibits the emergence of diverse kinship structures beyond the mere divergence of groups; this is considered unique to humans. The cooperation by marriage, as well as mating competition, leads to complex structures. We explained this in lines 567–575.

1) *The authors argue that the mate selection cycle that evolves in figure 2C represents generalized exchange. I agree that the mate selection cycle is described by the same* $C_m > 2$ *criterion, but the structure that evolves seems to be quite special. I would think that in empirically realized generalized exchange, individuals might choose mates from any clan (or at least from any one of a number of clans) other than their own. Perhaps the easiest way for this to arise would be a simple self-avoidance mechanism, where the phenotype* p *tells individuals which mates to avoid (e.g., members of their own family) rather than which to choose. In this case, it is not intuitive to me that this would lead to the emergence of exactly three (or indeed any) distinct clans. Avoidance (or negative preference) cannot arise in this model, and I am not suggesting the authors should change the model to allow it. But, it might be worth briefly discussing whether the form of generalized exchange that arises in this system really corresponds to that in empirical systems.*

>>
Anthropologists have observed the generalised exchange with $C_m > 2$ in which families have to choose a mate from a specific clan other than their own [4, 5]. The cases with $C_m = 3$ (or $4, 5, \cdots$) correspond to those observed in field studies. Whereas the system that prohibits only within-clan marriage does indeed exist, it is not covered by our model. We have stated this limitation in lines 292–296 and 583–586.

2) *Similar to my concerns about generalized exchange, I am not convinced that the system that the authors call an "incest structure" really reflects empirically realized incest structures. In the authors' incest structures, there are no clans at all, so it is hard to argue that mating occurs within clans. In figure 2A, the family traits and preferences do not line up on the* $t_1 = p_1$ *and* $t_2 = p_2$ *axes, so there does not seem to be a preference for incest within families. In fact, there might even be avoidance of incest within families — we cannot really tell without seeing the relationship between* $t_1 - p_1$ *and* $t_2 - p_2$*. Moreover, we might wonder if the incest structures that emerge in this model achieve the same trait topology as we might expect from empirical incest structures. For example, I can imagine a case where a preference for incest (i.e., mating within clans, not necessarily within families) allows clan traits to diverge in preference space, so reducing competition. In such cases, some forms of strong competition for mates might even favor the evolution of within-clan mating (e.g., if clans have a mechanism for suppressing internal competition, but not external competition). On the other hand, in the authors' model, where there is no mechanism for suppressing within-clan competition, clans with high incest might be destabilized by internal competition. Perhaps this could partly explain why empirical incest systems do not match the model predictions as well as the other kinship structures? (But, all of my thoughts here may result from my own poor understanding of empirical incest structures!)*

>>
The "incest structure" in our paper is one that allows mating within clans, not necessarily within families; therefore, it can be called endogamy. Hence, the rule preferring marriage within families is not discussed. In Fig. 2A, the variance of traits and preferences are only within the order of τ; and thus marriage within families can be conducted on a chance level. This situation evolves when d_c is large and d_m is small, which indicates a strong pressure for clustering and weak pressure for divergence. Indeed, with very small d_c and large d_m, families can line up on the $t_1 = p_1$ and $t_2 = p_2$ axes, which can be regarded as the preference for within-family marriages, which happens occasionally for small d_c. We show the example in Fig. S2.

3) *One interesting characteristic of this model is that (if I understand correctly), the traits and preferences we are studying are either strictly paternally or strictly maternally inherited, and there is no mixing or blending between them. So, an individual acquires traits (with error) from its paternal grandfather and its maternal grandmother, but there is no influence of its paternal grandmother or maternal grandfather*

(except of course that they influence whether the individual exists at all). There cases where this may be approximately true (e.g., surnames in some cultures), but others where some degree of blending may occur. The authors mention this in the discussion, albeit indirectly. However, it might help readers if the assumption of strict maternal and paternal trait acquisition were presented clearly very early in the manuscript. I think this would have helped me to understand the model more easily.

>>
Thank you for your suggestions. We clarified that we discuss the evolution of three types of descent systems in our revised manuscript, in lines 51–66 as follows. When children belong to the same clan as either their father or mother, the descent system is classified as patrilineal or matrilineal, respectively. In these cases, $C_d = 1$, and paternally or maternally inherited trait is significant for characterising clans. Conversely, when $C_d > 1$, children inherit cultural traits from both parents independently and have attributions different from either parent. When both paternally and maternally inherited traits are significant for characterising clans, the system is termed double descent. In the above cases, traits are assumed to be independently inherited through paternal and maternal lines. In some societies, however, people can choose either their father's or mother's traits to inherit in each generation (ambilineal descent), or they concern genealogical distance only (bilateral descent), which exceed the scope of our model [6, 7].

4) *I was, and remain to some degree, confused by time in this model. If a man and woman marry, then they form a new family, and that family (may) produce offspring in the next time step. The offspring have two time steps to marry, and if they remain unmarried then they are removed from the population. So, in this sense, time steps seem to correspond to generations. It is perhaps slightly odd that individuals can start families a full generation after they reach marriageable age, but this may be plausible for cultures with short generation times. But, is it true that the married pair is not removed from the population after they have reproduced, or in any subsequent time step? So, in effect, once married an couple can potentially persist and reproduce forever? If so, this seems very strange, and it might or might not matter for the model. A married pair that is 50 generations old might still be passing on traits and preferences from a much earlier form of its clan, and this might impede evolution, or might help a clan or society persist when it would otherwise disappear. Even if I am correct about this, I am not sure I would rerun the model to fix it (unless the authors had to rerun it for something else – then maybe it would be worth thinking about). But, it would help readers if the authors could explain time and the persistence of families in the model very clearly.*

>>
In our model, couples die soon after they reproduce, as stated in lines 230–234. Thus, your concerns will not occur in the present model.

Line-by-line comments:
Line 7: *This use of "attribution" was new to me, and I initially thought it meant "attributes." It might help non-specialist readers if the first use of attribution said something like "clan attribution (i.e., the clan to which they belong)."*

>>
Thank you for your suggestions for revision. We have added the explanation to attribution in lines 9–10.

Lines 28-47: *The formalism of describing kinship structures by (C_m, C_d) was also new to me, but is very important in this manuscript. I struggled to understand some of these ideas, particularly $C_d = 2$. I initially thought this meant that the children of mother A and father B would join some new clan to which neither parent belonged, but then that the grandchildren would somehow revert back to the clan of their grandfather B. But, I now think that the children somehow associate with different clans (sometimes the mother's and sometimes the father's) for different purposes, and that $C_d = 2$ indicates that the child half belongs to the father's clan (so the mean cumulative return time of a male descendent to the male progenitor's clan is 2). In any case, I think this could be more clearly explained. This is important because my failure to fully understand this idea initially later caused me to struggle with the idea of double descent in the model results.*

>>
We have revised the explanation of marriage and descent cycles in lines 21–33. Indeed, opinions are divided on double descent in cultural anthropology. Some regard the case with $C_m = 2$ as children belonging to several associations following each parent simultaneously, and others regard it as them belonging to a clan different from that of their parents. Our results can be interpreted from either of the viewpoints.

Lines 48-50: *I did not understand this sentence, possibly because I did not understand what "are referred to" meant.*

>>
We have clarified the explanation of double descent in lines 56–60 as follows: children inherit cultural traits from both parents independently and have attributions different from either parent.

Lines 113-116: *The introduction focuses on clans. When I reached these lines, I was confused about whether families or societies correspond to clans. It might help to tell the reader right here (or otherwise very early on) that clans are not initially created, but are instead an emergent property of the system.*

>>
Thank you for your very important suggestion. We have now explained that clans are emergent properties in our model in the introduction (lines 100–103).

Line 129: *I struggled with $|t_i - p_j|/\tau$, for an admittedly naïve reason. For mathematicians, the absolute value of a difference in vectors is a scalar, but in most computational languages it is still a vector. So, while the authors' notation is absolutely correct, I initially read this as being a vector. This caused me even more problems later when I was trying to understand how traits are transmitted. It might be worth reminding readers with poor mathematical training (like me!) that $|t_i - p_j|$ is just the Euclidean distance between the male trait combination and the female preference combination.*

>>
Thank you for this suggestion. We have now noted that $|\boldsymbol{t}^i - \boldsymbol{t}^j| = \sqrt{(t_1^i - t_1^j)^2 + (t_2^i - t_2^j)^2}$ represents Euclidean distance in lines 195–200.

Lines 150-151: *"At larger dc values, a small proportion of non-cooperators is crucial for survival." I initially read this to mean that, for some reason, families need at least a small number of non-cooperators to survive, but I think it means instead that they must have no more than a small number of non-cooperators if they are to survive. This could be stated more clearly.*

>>
Thank you for your suggestion. We clarified that d_c represents the increment of the death rate owing to non-cooperation, in lines 202–205.

Figures 2 and 3: *I am not sure I fully understand what is being represented here. In the legend to figure 3, the authors say they conducted "over 100 trials for each condition." But, what are the "conditions?" Presumably they are combinations of d_c, d_m, N_s, N_f, and μ. If so, what set of conditions was studied? I could probably guess by counting the squares in the figures (8×8?), but I would rather be told. And, how many is "over 100?" And, since each of these trials includes multiple societies, does that mean the real number of societies studied (since these are the levels at which kinship structure emerge) is > 5000? (I imagine we can assume that the > 50 societies generated by a single trial are effectively independent. I might be worth a quick check just to be sure.) Finally, in figure 2, are the societies shown just illustrative examples from the full set of societies and trials? That is, were they chosen because they look particularly good rather than in any sort of systematic or random way? (That is fine of course, but I would like to know!)*

>>
We conducted an evolutionary simulation 100 times for each pair of d_c, d_m values (values for N_s, N_f, and μ were set as constant in Fig. 3 and are varied in Fig. 5) and counted the frequencies with which each

structure evolved. Since the model involves stochasticity, the evolved societies can differ for each run. Hence, the total number of societies studied is 5,000 (indeed, many societies were replaced during the simulation, and the real number would be $\gg 5,000$). We cannot assume that 50 societies generated by a single trial are effectively independent, because fast-growing societies have many daughter societies.

We chose the examples that look good in Fig. 2, but they are typical. There may be some systematic way to choose "most typical" examples; however, exploring such procedures exceeds the scope of the present manuscript and has thus been excluded.

Lines 325-389: *I struggled with the empirical analysis more than with the model. Am I correct in believing that the authors looked at the set of SCCS variables and determined by knowledge of the system or by intuition (and hopefully before studying any correlations!!) whether each one was related to d_c or d_m? Then, after this, they asked whether each of these variables was correlated in the expected way (according to the authors' assessment of its role in d_c or d_m) with the empirical kinship structures, and this value is reported in table 3? Then, finally, they computed $\widetilde{d_c}$ and $\widetilde{d_m}$ values for each empirical population by averaging over the standardized and equally weighted variables that they believe contribute to d_c and d_m, and they plotted the empirical kinship structures as a function of $\widetilde{d_c}$ and $\widetilde{d_m}$ in figure 3B? If so, I am fine with all of this. But, I think the authors might make this easier to understand by explaining the assignment of SCCS traits to d_c or d_m first, and (if it is true) emphasizing that this was done before studying the correlations between traits and kinship structures. If the assignment of traits to d_c or d_m was not a priori, I still do not think it is fatal to the authors' argument, but it would influence how much confidence I would have in the assignments and in the empirical results for future work, so it is important to report.*

>>
Because there are no variables in SCCS that exactly correspond to d_c and d_m, we calculated the correlation between every SCCS variable and kinship structures first, without assigning them to d_c or d_m a priori. However, we hypothesised in advance that d_c can be related to the extent of social unity and external warfare, and d_m to the attitude toward adultery and the extent of internal warfare. By calculating the correlation for each variable and listing the variables in descending order in the absolute value of the correlation, we found those variables related to d_c and d_m in the above sense, in the top of the list (rather than the middle or bottom). We clarified this in lines 389–404. We further stated the necessity of collaboration with field studies to measure d_c and d_m directly, as a task for the future, in lines 594–596.

Line 690: *"Peter, T" should be "Turchin, P."*

>>
Thank you for your suggestion.

Reviewer # 3
0) *My thanks to the authors and editors for this patience on a review for this paper. As an evolutionary anthropologist interested in the evolution of human kinship I was pleased to be offered this paper to review. I think there are some excellent ideas here and I am delighted to see the use of ABMs alongside the empirical ethnographic data. However, I do not think it is suitable for PRSB for a few reasons I will explain briefly below.*

>>
Thank you for your evaluation and suggestions for improving our paper. Following your suggestions, we have accordingly updated our manuscript.

1) *The paper explores if various social, cultural and family-level parameters can produce kinship structures akin to the categories proposed by Levi-Strauss's alliance models. While very interesting and important to human kinship specialists, the paper is not motivated to be of interest to a wide evolution/biology/behaviour audience. Kinship is a complex topic in anthropology, and to be of broad interest beyond anthropologists the authors needed to make clear how understanding L-S's structures might shed more general light on human sociality, acting and cooperation. The structures themselves are taken-for-granted, and while they have been influential in anthropology since L-S's work in the 1950s, they are but one of a range of ways to categorise kinship arrangements in human societies. For a PRSB audience, we need a theoretical account*

of social behaviours grounded in evolutionary principles, and while the parameters in the ABM model could definitely be seen in that way the manuscript does not make the motivation for the model clear.

>>
Thank you for your suggestions. We have explained the importance of kinship in anthropology in lines 2–20 by stating that kinship has been considered one of the oldest and most frequently observed human social organisations [5, 7]. Moreover, kinship relationships between clans regulate other social relationships, such as cooperation or rivalry [8]. We also mentioned that kinship systems can be described in several ways by focusing on kinship terminologies or structures in lines 17–20.

Additionally, our results will interest readers as an example of the emergence of macroscopic structures from microscopic behaviours. We clarified this in lines 3–4 and 605–609. The structure formation by the interaction of agents will garner the general interest of biologists. How each kinship structure emerges depending on the pressures for cooperation and competition, because of the multi-level evolution, is consistent with theoretical biology and cultural evolutionary studies of social systems. Hence, this work will fit the broad readership of Proceedings B. Furthermore, similarity and differences of our model with sympatric speciation by mating competition, as noted by Reviewer 2 and stated in lines 567–575, will also capture the interests of the biologists.

2) *Despite very much being invested in understanding the work presented, I am sorry to say that I found this paper very hard to comprehend. From the outset it was not clear whether the existence of clans, or the existence of alliance structures, was the target of the model. When I tried to work my way through the specifics of the model I couldn't understand why there was multi-level population structure (families and societies) but no kinship organisation. There is strong evidence from our primate cousins that early hominids would have had some form of kinship structure (see work by e.g. Silk 2021 or Chapais 2008). Comparative cross-cultural work shows us that foraging societies, to the extent that they can provide a picture of the range of pre-Neolithic social structures, have flexibility built into their structures. But I wasn't sure what extent of human history the modelling was meant to provide an explanation for - the original emergence of alliance structures, or their repeated change over cultural evolutionary time? This last point speaks to a wider issue, which is that the manuscript does not have any ethnographic examples to illustrate the phenomena being explained. To speak to an audience beyond modellers, I think this is quite crucial.*

>>
Thank you for this detailed comment. Indeed, it is unreasonable to assume the group of families are without any social organisation; however, as stated in lines 237–242, our results do not qualitatively change against the choice of initial conditions. Even when we start the simulation by assuming some kinship structures as the initial state, the initial structure is soon reformed, and adaptive structure emerges depending on the parameter values of d_c and d_m. In our paper, we start from the neutral position, that is, assuming no rules at the initial state.

Our results would correspond to the repeated change of kinship structures over cultural evolutionary time. The formation of kinship structures requires several generations. Hence, its chronological records have been hardly described. Indeed, Lévi-Strauss demonstrated several examples of the sustenance of kinship structures. Cultural groups become divergent owing to population growth and internal conflict. Simultaneously, however, they are united by marital relationships. Even if some of the population is damaged, structures eventually recover within several generations [5]. We added this explanation in lines 469–482. Even though we may not precisely replicate the historical consequences, we can still explain how each kinship structure is sustained for each given condition.

3) *I reviewed from the perspective of a cross-cultural evolutionary anthropologist who studies kinship. I am familiar with and can follow ABMs, but I cannot attest to the rigour or quality of the simulation models and analytical approaches presented here – they might be exemplary but it would be remiss of me to claim I can judge their accuracy. One point of particular difficulty for me was the "cultural traits" that were crucial to the ABM. Families had these traits, and had preferences for them. Some examples were given – surnames, totems, and occupations – all of which are phenomena which may denote clan membership in some societies, and this felt circular. I was very unclear on whether these traits could be any kind of shared public signifier, and whether they could only be symbolic. Occupation, for example, signals information about resources as well as social desirability. I do think there is too much packed into*

this paper. It is the kind of work that would be better in another venue as a two-part paper Part 1: Model - Part 2: Empirical analyses, with ample space given to motivate the investigations and to describe and justify the parameters under study. The authors have made a valiant attempt at compressing a lot of work into a PRSB format but it is at the expense of clarity.

>>
The traits in our model represent any social features by which people can measure their cultural similarity, for example, surnames, occupations, or totems [9]. In our paper, we demonstrate that initially uniform traits gradually diverge to distinguish family groups. Hence, we modelled the process through which these traits will arise to denote clan membership in some societies. We added this explanation in lines 159–165.

We have intensively revised the manuscript and we believe it suits PRSB now.

4) *Finally, I felt on more familiar terrain with the final cross-cultural analyses in the paper, and I offer some specific remarks here which I hope the authors can use to improve their study.*
4-1) *The section "Classification of Kinship Structures" in the Methods could be much clearer. Interpreting the SM tables and values of variables requires deep specialist knowledge of kinship jargon and worked ethnographic examples would be more tractable.*

>>
Thank you for your suggestions. We have explained the method in detail in Supplementary Table S2, such that it would be more tractable for a broad readership. For example, we explained the identification of marriage rules as follows: Cousin preferences for mates provide the marriage rules between clans. Let us consider the woman with the largest genetic relatedness in the preferred clan: in patrilineal dual organisation, for example, a man in clan A is married to a woman in clan B. His father from A and his mother from B form a couple in A. By contrast, his mother's brother from B and his father's sister from A give birth to children in B. Hence, the woman with the largest genetic relatedness would be the daughter of father's sister and mother's brother. This is a symmetrical cross-cousin. If this was generalised exchange, his father's sister would marry a man in clan C, rather than A and B. Thus, the woman with the largest genetic relatedness would be the daughter of mother's brother, which is a matrilineal cross-cousin. The mating preferences for cross-cousins are well known in ethnographic studies [5, 10]. They also evolve in our simulation.

Moreover, we concisely explain our method by stating that we classified the kinship structures of each society by identifying the composition of clans and marriage and descent rules between them, in lines 375–377.

4-2) *Only 60/186 societies were included, because of the exclusive definitions of the four structures. The 186 societies in the SCCS are, to a first approximation, the most ethnographically well-described communities that anthropologists have studies, but L-S's framework can only account for kinship practices in a third of them. This weakens any claim of "universal anthropology" (the authors' choice of keywords).*

>>
Thank you for this comment. We have updated the identification of kinship structures by referring to kinship terminologies, in addition to cousin marriage preference, for determining marriage rules. Thus, out of 186 societies in SCCS, we identified 87 as incest structures, 14 as dual organisation, 33 as generalised exchange, and 12 as restricted exchange. Forty societies are excluded from the data analysis as their marriage rules prohibit within-clan (or family) marriage only. Subsequently, we analysed 146 societies, rather than 60. We have explained this in lines 377–388. Because our model does not cover the negative rules to exclude unpreferred mates without specifying preferrable mates, we could not cover all societies, as stated in lines 583–586. However, we can now cover 78% of societies in SCCS.

4-3) *Because only 60 were identified, I was less convinced by the "matching" data presented in Figure 4. This is not only because of the small sample size, but because of phylogenetic and spatial autocorrelation effects. These still exist in the SCCS, despite wishful-thinking claims to the contrary.*

>>

We understand the limitation of the data used for statistical analysis. Nevertheless, we could analyse 146 (rather than 60) societies that spread out globally, as shown in Fig. S1. Accordingly, we have revised Fig3, to increase the data points. We stated the necessity of phylogenetic comparative analysis to control statistical non-independence owing to shared ancestry as a limitation in lines 598–600. However, Figure S1 shows that each structure is spread out globally, without a clear spatial pattern. Especially the large variation of kinship structures in South Asia or Oceania, for example, suggests a small autocorrelation. This suggests that the kinship structures in each region are achieved by cultural adaptation, rather than cultural transmission, which must be further investigated by phylogenetic comparative analysis in the future. We have added this explanation in lines 384–388.

4-4) *The authors then looked for explanatory variables for the kinship structures (line 340): "we conducted Spearman's rank correlations between the SCCS variables and the structures." I think what they have done here is to do a massive set of correlations of all 2000 variables in the SCCS with the target kinship structures. They state that more sophisticated approaches would be desirable but data insufficiencies preclude this approach. Nonetheless they present a list of so-called explanatory variables that again do not have any controls for historical relatedness or geographical proximity. I have no objection to brute-force exploratory analyses, but this is one area where some theoretically-motivated predictions would have been more desirable than the list in Table 3 with post-hoc considerations.*

>>
Thank you for your suggestions. We have added the limitation concerning phylogenetic comparative analysis as described above.

References

[1] Luigi Luca Cavalli-Sforza and Marcus W Feldman. *Cultural transmission and evolution: A quantitative approach.* Princeton University Press, 1981.

[2] Carmela Rosalba Guglielmino, Carla Viganotti, Barry Hewlett, and Luigi Luca Cavalli-Sforza. Cultural variation in africa: Role of mechanisms of transmission and adaptation. *Proceedings of the National Academy of Sciences*, 92(16):7585–7589, 1995.

[3] Ulf Dieckmann and Michael Doebeli. On the origin of species by sympatric speciation. *Nature*, 400(6742):354–357, 1999.

[4] Edmund Ronald Leach. *Political Systems of Highland Burma.* Beacon Press, 1954.

[5] Claude Lévi-Strauss. *Les structures élémentaires de la parenté.* Paris: Presses Universitaires de France, 1949.

[6] Claude Lévi-Strauss. The future of kinship studies. *Proceedings of the Royal Anthropological Institute of Great Britain and Ireland*, (1965):13–22, 1965.

[7] E.R. Service. *Primitive Social Organization: An Evolutionary Perspective.* Random House studies in anthropology. Random House, 1962.

[8] Robin Fox. *Kinship and marriage: An anthropological perspective*, volume 50. cambridge university press, 1983.

[9] Claude Lévi-Strauss et al. *La pensée sauvage*, volume 289. Plon Paris, 1962.

[10] George P Murdock and Douglas R White. Standard cross-cultural sample. *Ethnology*, 8(4):329–369, 1969.

Appendix C

Point-by-point responses: “Emergence of Kinship Structures and Descent Systems: Multi-level Evolutionary Simulation and Empirical Data Analysis” (RSPB-2021-2641)

Referee: 2
Comments to the Author(s).
I have just read the revision of manuscript RSPB-2021-1136, now numbered RSPB-2021-2641. I believed this was an interesting and valuable contribution in the initial round of review, and I still believe that now. Moreover, I believe the manuscript itself has improved in revision. I have only minor suggestions for further improvement. Of course, the authors may take or leave these as they see fit.

>>
Thank you for your positive evaluation and suggestions for revision. Following your suggestions, we have accordingly updated the paper to provide a better explanation.

Line 2: “form the basic units of society. . . ”: Should “form” be “shape?” To me, “form” implies that marriage and descent somehow constitute the basic units of society (ie, families), but I think the authors means that they determine how families arise and behave.

>>
Thank you for the suggestion. We have updated the manuscript accordingly.

Lines 8-9: “Marriage and descent relationships are, thus, determined by clan attributions”: Maybe “Marriage and descent relationships, and also kinship, are determined by clan attributions?” For me as a biologist, kinship implies genetic relatedness, but I think that is not necessarily true here, and it took me a while to understand that members of the same clan who are not genetically related are still “kin” in this study. Or, if I am wrong about this, then maybe a very clear definition of “kinship” would help!

>>
Thank you for a helpful suggestion. We added the explanation for “kinship” as relationships including marriage, descent, and cultural relatedness in lines 3–4. Additionally, we have updated the explanation in lines 8–9 accordingly.

Line 55: Should be “. . . and the paternally. . . ”

>>
Thank you for the suggestion. We have updated the manuscript accordingly.

Line 69: Should “dominant over” be “more common than?” For me, “dominant over” implies that when two things interact, the dominant one will somehow prevail. But, it is not clear to me that there is any interaction or prevailing in the empirical systems. (Perhaps there is in the model, but that is not what the authors are describing here.)

>>
Thank you for the suggestion. We have updated the manuscript accordingly.

Line 72-73: “distinction of cultural associations”: I do not understand what this means.

>>
Thank you for the suggestion. We have updated this to “this perspective ignores that people are categorised as clans by symbolic traits inherited through some descent lines” for further clarity.

Line 92: Is “indigenous” the right word here? Maybe “self-organising?”

>>
Thank you for the suggestion. However, in this line, we want to state that we have modeled the family behaviour that are common in many traditional indigenous societies. Hence, we keep using “indigenous”

with adding the explanation in lines 93–94.

Line 124: I think "unveil" is too strong a word here. Maybe "suggest"

>>
Thank you for the suggestion. We have updated the manuscript accordingly.

Line 157: "by eliminating it": Maybe "by eliminating lineages?" It was not 100% clear to me what "it" referred to in this sentence.

>>
Thank you for the suggestion. We have updated the manuscript accordingly.

Line 189: Should "supposed" be "assumed?" That is, this is a specific assumption of this model, and not a consensus opinion about empirical systems based on previous research, right?

>>
Thank you for a helpful suggestion. As you say, it is mere our assumption. We have updated the manuscript accordingly.

Figure 1B: This figure confused me a little. Would there not also be competition between two families with the same preference AND the same trait value? If so, I would expect an arrow from the top left family that loops back to itself. But, if each circle represents just one family rather than a class of families, then maybe this would be even more confusing, because the authors do not want to imply that families compete with themselves. Also, I was a little confused by the grid-like layout of the families in 1B. This seems to imply that traits and preferences are discrete, but in practice (at least at the outset of the model, which is what the authors are describing here) they are continuous. All in all, I am not sure whether figure 1B makes the model clearer or less clear. One thing that might help is if the authors could be very clear at this point in the text (maybe the paragraph beginning on line 206) that every family is both a friend (based the similarity of their traits, or the match between their traits and preferences) and a rival (based on the similarity of their preferences) to every other. This becomes clear later in the manuscript, but would have helped me here.

>>
Thank you for a helpful suggestion. Indeed, it is important to note that the same family can be a rival and kin/mate at the same time. We added this explanation in the caption of Figure 1B. As for the figure, it shows four families (but not classes of families), so it will be confusing if we add an arrow from the top left family that loops back to itself. Besides, families may seem to have discrete traits in the figure, but we think readers can find they are continuous ones from other figures (such as Figure 2) and the explanation (such as lines 173–180). Hence, we keep using grid-like layout of families.

Line 240: "without introducing any rules": I might say "starting from an initially homogenous state." After all, there are MANY rules (eg, about how families compete, how and when marriages form, etc).

>>
Thank you for the suggestion. We have updated the manuscript accordingly.

Line 255-256: "Siblings belonged to the same cluster." Maybe specify "at birth," since this need not be true after marriage? (This probably seems pedantic, but it really did confuse me for a minute, and I wondered if maybe the authors were referring to sibling families.)

>>
Thank you for a helpful suggestion. As you say, siblings of different sex belong to different clusters after marriage. Hence, we have updated the manuscript accordingly.

Line 285: Perhaps specify that a matrilineal descent system evolves in this particular simulation, but a patrilineal system might evolve in others? (I think this becomes clearer later, but might help readers here.)

>>
Thank you for a helpful suggestion. As you noted, a patrilineal system can evolve under the same parameter values where a matrilineal system evolves. To clarify it, we explained as below in lines 302–304. Indeed, various kinship structures and descent systems evolve even under the same environmental parameter values d_c and d_m. However, statistically, evolved kinship structures and descent systems depend on parameter values.

Lines 364-367: I was confused here by whether the authors were referring to the model or to real systems. I think the word "realistically" is meant to make that clear, but it did not fully work for me. Maybe "In real systems. . . "

>>
Thank you for the suggestion. We have updated the manuscript accordingly. Figure 5 and its explanation are currently included in Supplementary Material due to page length limmit.

Lines 380-382: "Forty societies were . . . marriage only." Why are these societies not examples of generalised exchange? From the introduction I thought they should be.

>>
Thank you for the suggestion, but they are not categorised as generalised exchange. When there solely exists the rule to prohibit within clan (or family) marriage, kin groups never form any stable structure. Hence, the rule to specify preferable mates and that to prohibit within clan (or family) marriage only are distinguished in the kinship studies. Here, we focus on the former. We explained that those societies where within clan (or family) marriage are excluded from the analyses because they do not specify any preferable mates in lines 371–373.

Line 570 "mating competition or niche construction." I might say "resource competition and evolved mate preferences." The authors might want to have a look at Bolnick and Doebli 2003 (Evolution), which includes an interesting case where disassortative mating (ie, a preference for mates with traits dissimilar to one's own) leads to speciation (see figure 8). This seems similar to the restricted exchange with double descent that the authors obtain here. I think most biologists agree that this is an implausible outcome in speciation models, but as the authors explain, human cultural evolution does not follow exactly the same rules. Personally I find it interesting that an idiosyncratic mechanism in Bolnick and Doebeli's work might reflect an important mechanism in this new context.

>>
Thank you for the helpful suggestion and introducing us an interesting paper. Indeed, Figure 8 in Bolnick and Doebli 2003 (Evolution) looks similar to the restricted exchange in our paper. However, they are fundamentally different. The result of Bolnick and Doebli shows two species with sexual dimorphism, where the parents of a species (namely) A do not bare children of the other species B. Hence, although species A and B interact ecologically, they do not form kinship relationships. However, in the restricted exchange in our paper, four clusters of families are united by kinship relationships as a couple of a man in clan A_1 and a woman in B_2 give birth to children in A_2, for example. Therefore, results in Bolnick and Doebli 2003 (Evolution) are apparently similar to ours but actually different. We think it will be confusing to explain these, so we do not refer to this paper in the manuscript.

Lines 580-583: "As population size . . . is prohibited." Again, this is in real systems, right? Perhaps specify just to avoid any confusion.

>>
Thank you for a helpful suggestion, and yes it is. We have updated the manuscript accordingly.

Line 611-612: "we have demonstrated that various kinship structure emerge": In the model, correct? If so, perhaps specify. As the authors explain above, more work needs to be done to determine whether these same mechanisms have really shaped human kinship structures. To me, much of the value of this paper is that it invites this further work!

>>

Thank you for a helpful suggestion. In this part, we explain that the combination of theoretical simulation and empirical data analysis will contribute to building the general theory in anthropology. Hence, the statements is not for the model only. Although we have tested the environmental dependence of kinship structures, we are currently unable to test whether they have emerged as we proposed. Therefore, we have updated the manuscript to “combined with theoretical simulations of a simple constructive model and empirical data analyses, we have explained the dependence of various kinship structures around the world upon the degrees of cooperation and avoidance of competition” in lines 601–603.
